# Daily routine disruptions and psychiatric symptoms amid COVID-19: a systematic review and meta-analysis of data from 0.9 million individuals in 32 countries

**DOI:** 10.1186/s12916-024-03253-x

**Published:** 2024-02-02

**Authors:** Huinan Liu, Tiffany Junchen Tao, Selina Kit Yi Chan, Jeremy Chi Him Ma, Abby Yan Tung Lau, Ernest Tsun Fung Yeung, Stevan E. Hobfoll, Wai Kai Hou

**Affiliations:** 1 Centre for Psychosocial Health, The Education University of Hong Kong, Hong Kong SAR, China; 2 Department of Special Education and Counselling, The Education University of Hong Kong, Hong Kong SAR, China; 3 University of California Irvine, Irvine, CA USA; 4 STAR Consultants-STress, Anxiety and Resilience, Salt Lake City, UT USA; 5 Department of Psychology, The Education University of Hong Kong, Tai Po NT, 10 Lo Ping Road, Hong Kong SAR, China

**Keywords:** Daily routines, Mental disorders, Social and environmental determinants, COVID-19, Large-scale disasters

## Abstract

**Background:**

There is currently a deficit of knowledge about how to define, quantify, and measure different aspects of daily routine disruptions amid large-scale disasters like COVID-19, and which psychiatric symptoms were more related to the disruptions. This study aims to conduct a systematic review and meta-analysis on the probable positive associations between daily routine disruptions and mental disorders amid the COVID-19 pandemic and factors that moderated the associations.

**Methods:**

PsycINFO, Web of Science, PubMed, and MEDLINE were systematically searched up to April 2023 (PROSPERO: CRD42023356846). Independent variables included regularity, change in frequency, and change in capability of different daily routines (i.e., physical activity, diet, sleep, social activities, leisure activities, work and studies, home activities, smoking, alcohol, combined multiple routines, unspecified generic routines). Dependent variables included symptoms and/or diagnoses of mental disorders (i.e., depression, anxiety, post-traumatic stress disorder, and general psychological distress).

**Results:**

Fifty-three eligible studies (51 independent samples, 910,503 respondents) were conducted in five continents. Daily routine disruptions were positively associated with depressive symptoms (*r* = 0.13, 95% CI = [0.06; 0.20], *p* < 0.001), anxiety symptoms (*r* = 0.12, 95% CI = [0.06; 0.17], *p* < 0.001), and general psychological distress (*r* = 0.09, 95% CI = [0.02; 0.16], *p* = 0.02). The routine-symptom associations were significant for physical activity, eating, sleep, and smoking (i.e., type), routines that were defined and assessed on regularity and change in capability (i.e., definition and assessment), and routines that were not internet-based. While the positive associations remained consistent across different sociodemographics, they were stronger in geo-temporal contexts with greater pandemic severity, lower governmental economic support, and when the routine-symptom link was examined prospectively.

**Conclusions:**

This is one of the first meta-analytic evidence to show the positive association between daily routine disruptions and symptoms of mental disorders among large populations as COVID-19 dynamically unfolded across different geo-temporal contexts. Our findings highlight the priority of behavioral adjustment for enhancing population mental health in future large-scale disasters like COVID-19.

**Supplementary Information:**

The online version contains supplementary material available at 10.1186/s12916-024-03253-x.

## Background

Decades of converging evidence has revealed how the etiology of mood disorders is attributable to biological underpinnings of social rhythm dysregulations [1] and how family routines provide an environment that is conducive to individual members’ positive psychosocial adjustment [2]. However, it was not until the outbreak of the unprecedented COVID-19 pandemic that daily routine disruptions were widely recognized as an important, universal determinant of poorer population mental health [3, 4]. Under the prevailing global impact of the pandemic and associated infection control rules, many studies have investigated the extent to which disruptions to daily routines could be positively related to mood disorders or their subclinical symptoms, suggesting daily routine disruptions as a tipping point for mental disorders [3, 4].

There is currently a deficit of knowledge about how to define, quantify, and measure different aspects of daily routine disruptions amid large-scale disasters like COVID-19, not to mention which psychiatric symptoms were more related to the disruptions. In addition, a growing body of research has suggested the social determinants of the intimate associations of COVID-19 infection, social distancing, and lockdown with disrupted daily routines and heightened psychiatric symptoms. Individuals with lower levels of or lower access to socioeconomic resources were more likely to experience disruptions in their daily routines or practice unhealthy behaviors, which were positively related to higher levels of psychological distress or psychiatric symptoms [5–9].

Little is known about whether and how the associations of routine disruptions with mental disorders differ across types and contexts. A handful of systematic reviews and meta-analyses have summarized the associations of mental health with specific daily activities, including physical activity [10, 11], dietary behaviors [12], sleep [13], social media use [13], social isolation [14], and working from home [15]. Because the global impact of COVID-19 was present over an unprecedented extended period of time while pandemic severity and infection control rules varied drastically across regions, there is a need to identify the spatiotemporal factors that impact the associations between daily routine disruptions and symptoms/diagnosis of mental disorders.

This study aims to conduct a systematic review and quantitative synthesis of how different aspects of routines as disrupted by COVID-19 could be related to symptoms and/or diagnoses of common mental disorders. We also sought to examine how the routine-symptom associations could vary across different populations, contexts, time periods, geographic locations, pandemic severity, pandemic policy responses, and study designs. We tested the following two hypotheses based on the central assumptions of the Social Zeitgeber Model, Drive to Thrive (DTT) theory, and the Family Routines Framework [1, 2, 16] that routine disruptions relate to higher psychiatric symptoms:

- *Hypothesis 1*. Disruptions to daily routines will be positively associated with psychiatric symptoms.
- *Hypothesis 2*. The positive associations between routine disruptions and psychiatric symptoms will be moderated by various factors, including types and definitions/assessments of routines, types of mental disorders, sociodemographics, spatiotemporal dimensions of COVID-19, and study designs.

## Methods

### Search strategy and selection criteria

This systemic review and meta-analysis followed Preferred Reporting Items for Systematic Review and Meta-Analysis (PRISMA) guidelines [17] and was originally registered in PROSPERO (CRD42023356846). Four databases (e.g., MEDLINE, PubMed, PsycINFO, and Web of Science) were searched for primary studies from inception up to April 6th 2023, using a combination of three categories of keywords: *COVID-19*, *mental health*, and *daily routines*. Supplementary Material 1 outlines the detailed search strategies.

Inclusion criteria were (1) empirical studies conducted during the COVID-19 pandemic; (2) studies using quantitative self-report of daily activities in terms of regularity, change in frequency, or change in capability since COVID-19; and (3) studies using at least one psychometrically validated quantitative measure of mental disorders (i.e., symptoms and/or diagnoses). Studies were excluded if (1) any one or more of the three key components, namely COVID-19, daily routines, and psychiatric symptoms/diagnosis, were absent; (2) effect size was not reported; (3) symptoms/diagnoses of mental disorders were not assessed using validated psychometric instruments; or (4) the findings were not published in English peer-reviewed journals. All stages of data extraction were checked to ensure accuracy and agreed upon by HL, TJT, and WKH. To begin with, titles and abstracts were independently screened by a group of four reviewers (SKYC, AYTL, JCHM, ETFY). Studies with inconsistent assessment of their eligibility were retained for the next stage of screening. For the second stage, four independent reviewers/authors (HL, TJT, SKYC, WKH) were involved in the data extraction process. Eligibility of each included article was double-checked by a second reviewer from the four in the second stage [18]. Any disagreements were resolved through discussion and reiteration of the extraction among the authors.

### Data extraction and quantitative synthesis on the effect sizes

The following data were extracted from eligible studies by independent reviewers (): sociodemographics (i.e., sample size, age, gender, marital status, education, employment, country of origin, and physical comorbidity), study design (i.e., cross-sectional vs. prospective design, durations of prospective follow-ups), COVID-19-related variables (i.e., number of months since COVID-19 break, COVID-19 monthly cumulative incidences per million, COVID-19 monthly cumulative deaths per million, and four COVID-19 policy indices defined by the Oxford Covid-19 Government Response Tracker (OxCGRT)) [19]. Information on the monthly cumulative incidences/deaths per million was extracted from official websites, while four COVID-19 policy indices were extracted from Oxford Covid-19 Government Response Tracker (OxCGRT; [19]). The four indices included COVID-19 government response index (i.e., strength of lockdown, health, and economic support policies), COVID-19 containment and health index (i.e., strength of lockdown and health policies), COVID-19 stringency index (i.e., strength of lockdown policies), and COVID-19 economic support index (i.e., strength of economic support policies). We also considered characteristics of daily routines (i.e., type, definition, and assessment; internet-based or not; validated measurement or not), and type of symptoms and/or diagnoses of mental disorders (i.e., depression, anxiety, post-traumatic stress disorder (PTSD), and general psychological distress). Type of routines included physical activity, eating, sleep, social activities, leisure activities, work/studies, and home activities [16, 20]. Other routines were categorized as either combined multiple routines (i.e., more than one type of routines) or unspecified generic routines (i.e., no further information on types). Definition of routines referred to regularity, change in frequency, and change in capability. Routine disruptions were reflected by high scores of changes in regularity, frequency, or capability, which were expected to lead to more psychiatric symptoms. The detailed coding sheet is shown in Supplementary Material 2.

Pearson product-moment correlation coefficient (*r*) was used as the effect size metric of interest to indicate the zero-order associations between daily routine disruptions and mental disorders. Other formats of effect sizes such as un/standardized regression coefficients, odd ratios, and *χ*^2^ were converted into correlations using the formula summarized in Supplementary Material 3. To address the issue of effect size dependency, effect sizes were averaged if (1) the original paper analyzed multiple levels of the same routine, or (2) multiple effect sizes were reported for the same type of routine with the same characteristics (i.e., definition, internet-related or not, and assessment method). To pool the effect sizes, correlation coefficients were then transformed into normally distributed Fisher’s *Zr* to adjust for skewed distributions. Random effect models were used to test the study hypotheses unless otherwise stated. All computations were performed in the R platform using metafor package [21, 22].

### Quality assessment and publication bias

The 20-item AXIS tool was used to assess study quality on three dimensions: quality of reporting, quality of study design, and possible introduction of bias [23]. Total and the three subscale scores were calculated for each study, with high scores indicating better quality (Supplementary Material 4). Publication bias was visualized by funnel plots and then examined by Egger’s regression test for funnel plot asymmetry and corrected by the Duval and Tweedy trim-and-fill method. Failsafe-*N* test was conducted to determine the number of missing studies that would turn the pooled effect size insignificant.

### Subgroup analysis

*Q*-tests in subgroup analysis and meta-regression were performed to test potential categorical and continuous moderators, respectively: characteristics of routines (type of routines, definition and assessment of routines, internet-based or not, validated measure(s) or not), type of psychiatric symptoms, population characteristics (i.e., country-level income, percentage of females, percentage of secondary education or below, percentage of non-married statuses, percentage of non-employed statuses, percentage of ICD-defined physical comorbidity), contextual and spatiotemporal features of COVID-19 (i.e., continent, number of months since COVID-19 outbreak, COVID-19 monthly incidences per million, COVID-19 monthly deaths per million, COVID-19 government response index, COVID-19 containment and health index, COVID-19 stringency index, COVID-19 economic support index), and study design (i.e., total scores on AXIS, cross-sectional or prospective associations between routines and outcomes, follow-up duration (months) for prospective studies).

## Results

### Included studies

**Fig. 1:**
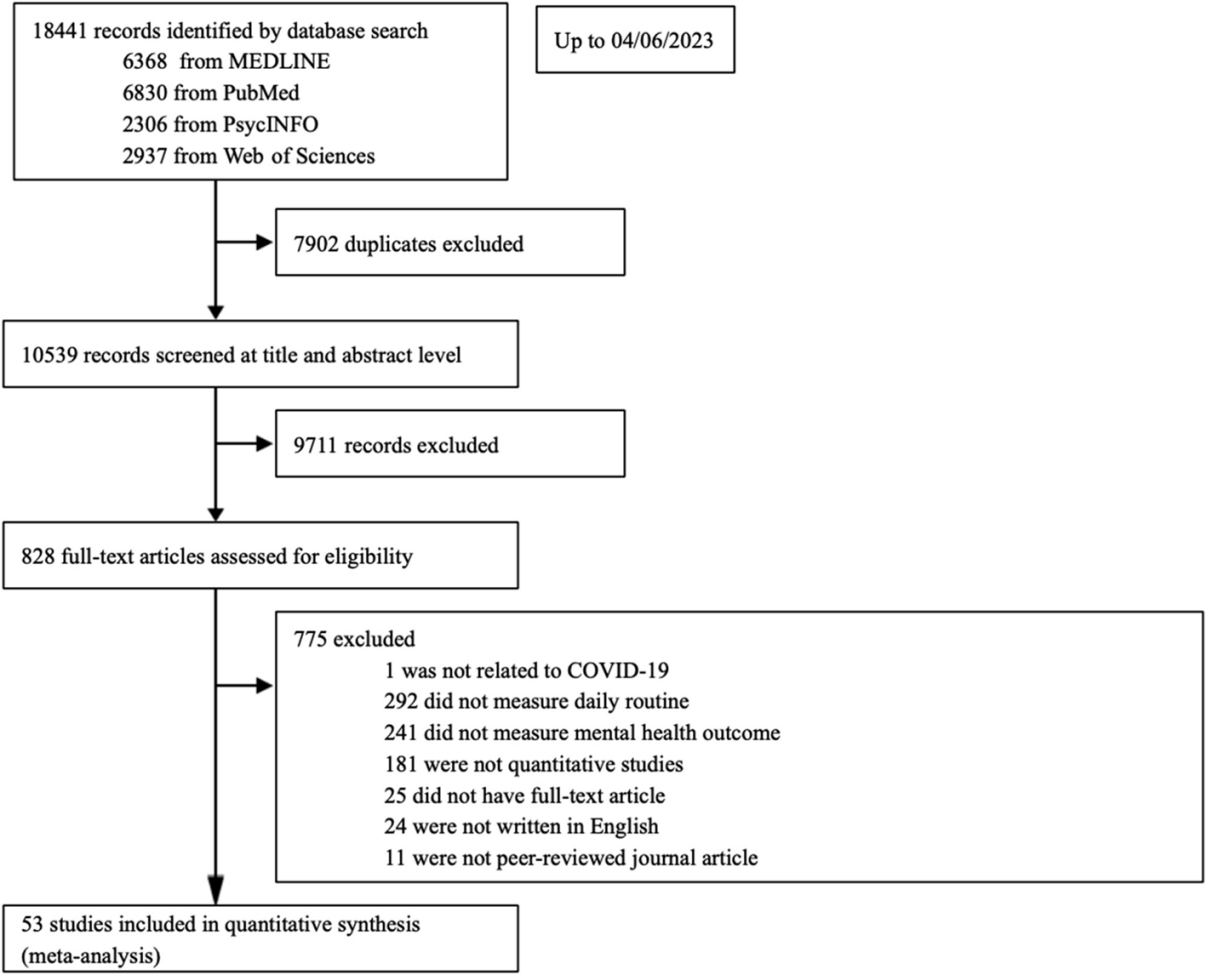
PRISMA flowchart

Figure 1 shows the PRISMA flowchart elaborating on the detailed selection process. The present review included 53 eligible articles from 51 independent samples of 910,503 respondents from 32 regions across five continents: 24 studies in Asia (China, Hong Kong SAR, Japan, Jordan, Pakistan, Saudi Arabia, Singapore, and South Korea); 20 in Europe (Belgium, France, Germany, Greece, Ireland, Italy, Norway, Poland, Spain, Sweden, Turkey, and UK); three in North American (USA); two in South America (Chile and Colombia); and one in Oceania (Australia). Data were collected from the acute phase of COVID-19 to 2.5 years after the initial outbreak (i.e., mid-2022). Across the entire study period, COVID-19 monthly incidence (per million) ranged from 0.98 to 58,642.57 (*M* = 3670.40, *SD* = 9981.34), COVID-19 monthly death (per million) ranged from 0 to 571.470 (*M* = 56.51, *SD* = 112.85), and COVID-19 government response index (on a scale of 0–100) ranged from 36.980 to 81.770 (*M* = 64.112, *SD* = 9.718). Supplementary Material 5 summarizes the bibliographical referencing and descriptive information of all included studies [16, 20, 24–78].

Table 1 illustrates the descriptive statistics of the 53 eligible studies. Respondents aged between 13 and 71 years (*M* = 39.75, *SD* = 16.23). The proportion of females ranged from 42–100%, and respondents with secondary education or below ranged from 0 to 100%. About 5–98% were non-married (i.e., proportion of sample that were not married or in stable relationship), 0–93% non-employed (i.e., proportion of sample that were not employed), and 20–100% with at least one ICD-defined physical comorbidity. Most studies (84.31%) were conducted in high-income countries.

**Table 1:** Descriptive statistics of 53 included studies (51 independent samples)

Characteristics	Samples with characteristics, No. (%)
*Population demographics*	
Sample size, mean (SD) [range]	17,853.00 (61,618.38) [40–379, 875]
Mean age, mean (SD) [range] (9 samples “No report”)	39.75 (16.23) [13.29–71.03]
Age range (30 samples “No report”)	10–99
Proportion (%) of respondents with particular demographic characteristics, mean (SD) [range]	
Non-male	65.00 (15.38) [42.06–100]
Without tertiary education (21 samples “No report”)	39.15 (34.27) [0–100]
Non-married (30 samples “No report”)	44.47 (17.95) [4.98–98.32]
Non-employed (25 samples “No report”)	37.87 (27.95) [0–93.26]
ICD-defined physical comorbidity (42 samples “No report”)	51.74 (22.79) [19.66–100]
Country-level income	
High	43 (84.31%)
Middle	8 (15.69%)
*Coronavirus-2019 contextual and spatiotemporal features*	
Continent	
Asia	24 (47.06%)
Europe	20 (39.22%)
Africa	0 (0.00%)
North America	3 (5.88%)
South America	2 (3.92%)
Oceania	1 (1.96%)
Multiple continents	1 (1.96%)
Number of months since COVID-19 outbreak, mean (SD) [range]^a^ (4 samples “No report”)	10.11 (6.85) [3–29]
COVID-19 monthly incidence (per million), mean (SD) [range]^a^ (6 samples “Data not retrievable”)	3,670.40 (9,981.34) [0.984–58,642.574]
High	22 (41.51%)
Low	22 (41.51%)
COVID-19 monthly death (per million), mean (SD) [range]^a^ (6 samples “Data not retrievable”)	56.51 (112.85) [0–571.470]
High	22 (41.51%)
Low	22 (41.51%)
COVID-19 government response index, mean (SD) [range]^b^ (6 samples “Data not retrievable”)	64.112 (9.718) [36.980–81.770]
High	22 (41.51%)
Low	22 (41.51%)
COVID-19 containment and health index, mean (SD) [range]^b^ (6 samples “Data not retrievable”)	62.798 (10.123) [36.907–84.520]
High	22 (41.51%)
Low	22 (41.51%)
COVID-19 stringency index, mean (SD) [range]^b^ (6 samples “Data not retrievable”)	65.087 (15.590) [35.190–90.396]
High	22 (41.51%)
Low	22 (41.51%)
COVID-19 economic support index, mean (SD) [range]^b^ (6 samples “Data not retrievable”)	73.305 (26.480) [8.333–100]
High	22 (41.51%)
Low	22 (41.51%)
*Psychiatric symptoms*	
Depressive symptoms	34 (66.67%)
Anxiety symptoms	24 (47.06%)
Post-traumatic stress disorder (PTSD) symptoms	4 (7.84%)
Depressive and anxiety symptoms	4 (7.84%)
General psychological distress^C^	12 (23.53%)
*Daily routine disruptions*	
*Category*^d^	
Primary routines	19 (37.25%)
Secondary routines	25 (49.02%)
*Type*	
Physical activity	12 (23.53%)
Eating	5 (9.80%)
Sleep	11 (21.57%)
Social activities	11 (21.57%)
Leisure activities	8 (15.69%)
Work/Study	9 (17.65%)
Home activities	7 (13.73%)
Smoking	2 (3.92%)
Alcohol	3 (5.88%)
Combined multiple routines	8 (15.69%)
Unspecified generic routines	16 (31.37%)
*Definition*	
Regularity	24 (47.06%)
Frequency change	22 (43.14%)
Capability change	10 (19.61%)
*Internet-related or not *	
No	51 (100.00%)
Yes	8 (15.69%)
*Assessment method*	
Validated scale	17 (33.33%)
Non-validated scale	36 (70.59%)
*Study feature*	
Study quality, mean (SD) [range] (Based on 50 studies)	14.75 (1.82) [9–18]
Observational or experimental design	
Observational	51 (100.00%)
Experimental	0 (0.00%)
Cross-sectional or prospective design	
Cross-sectional	48 (94.12%)
Prospective	4 (7.84%)
Follow-up duration (weeks), mean (SD) [range] (1 sample “No report”)	7 (3.74) [3–12]

The detailed information of individual studies is available in Supplementary Material 5

One sample had both cross-sectional and prospective effect sizes [30]. One sample had both high- and middle-income countries [42]

^a^The unit is monthly cumulative per million individuals. Information was extracted from official websites. “High” and “Low” categories were generated based on median split

^b^The unit is monthly average score. Information was extracted from Oxford Covid-19 Government Response Tracker (OxCGRT; [19]). “High” and “Low” categories were generated based on median split

^c^“General psychological distress” included distress (e.g., “Kessler Psychological Distress Scale–6 (K6)”) and stress (e.g., “Depression Anxiety Stress Scale-21 (DASS-21) Stress Subscale”)

^d^Primary routines included eating, sleep, and home activities; Secondary routines included physical activity, leisure activities, social activities, and work/studies [16, 20]

Psychiatric symptoms included depressive symptoms (66.67%), anxiety symptoms (47.06%), PTSD symptoms (7.84%), depressive and anxiety symptoms (7.84%), and general psychological distress (23.53%). Types of routines included unspecified generic routines (31.37%), physical activity (23.53%), sleep (21.57%), social activities (21.57%, including both offline and online interaction with family, friends, neighbor, and health professionals), work/study (17.65%), leisure activities (15.69%, including screen time, personal care, going out, and interests), combined multiple routines (15.69%), home activities (13.73%, including childcare, elder care, household activities, personal hygiene, and tidiness), eating (9.80%), alcohol (5.88%), and smoking (3.92%). Most were measured using non-validated measures (70.59%). A total of 47.06% defined and assessed routines as regularity, 43.14% as change in frequency, and 19.61% as change in capability. About 10% of the routines were internet-based. Most studies adopted cross-sectional design (94.12%). The average score of study quality was 14.75 (*SD* = 1.82).

### Associations between daily routine disruptions and mental disorders

Overall, the positive association between daily routine disruptions in aggregate and mental disorders was significant (*r* = 0.11, 95% CI = [0.07; 0.14], *p* < 0.001). Pooled effect sizes suggested that routine disruptions were significantly positively associated with depressive symptoms (*r* = 0.13, 95% CI = [0.06; 0.20], *p* < 0.001), anxiety symptoms (*r* = 0.12, 95% CI = [0.06; 0.17], *p* < 0.001), and general psychological distress (*r* = 0.09, 95% CI = [0.02; 0.16], *p* = 0.02). Routine disruptions were not associated with PTSD symptoms (*r* = 0.03, 95% CI = [− 0.09; 0.15], *p* = 0.56) and combined depressive and anxiety symptoms (*r* = 0.01, 95% CI = [− 0.01; 0.02], *p* = 0.38). Pooled effect sizes of the associations between daily routine disruptions and psychiatric symptoms are summarized in Table 2. Forest plots showing effect sizes from individual studies are listed in Supplementary Material 6.

**Table 2:** Pooled effect sizes of the association between daily routine disruptions (combined across types) and psychiatric symptoms (53 studies, 51 independent samples)

Outcome	*k*	Pooled *r* [95% CI]	*p*	*I*^2^ (%)	*Q*
Mental health (overall)	**145**	**0.11 [0.07; 0.14]**	** < 0.001**	**99.9**	**246,941.59**
Depressive symptoms	**62**	**0.13 [0.06; 0.20]**	** < 0.001**	**100.0**	**238,617.20**
Anxiety symptoms	**38**	**0.12 [0.06; 0.17]**	** < 0.001**	**98.6**	**2715.86**
﻿Post-traumatic stress disorder (PTSD) symptoms	12	0.03 [-0.09; 0.15]	0.56	98.9	1010.73
Depressive and anxiety symptoms	6	0.01 [-0.01; 0.02]	0.38	0	0.44
General psychological distress	**27**	**0.09 [0.02; 0.16]**	**0.02**	**98.3**	**1536.27**

Definitions

*k* = Number of effect sizes. Bold texts indicate significant results. The detailed forest plots presenting effect sizes from individual studies are available in Supplementary Material 6

Risk of publication bias is visualized in funnel plots (Supplementary Material 7). Publication bias was detected between routine disruptions and anxiety symptoms (Egger’s regression intercept =  − 4.45, 95% CI = [− 7.34, − 1.74], *t* =  − 3.18, *p* < 0.01), PTSD symptoms (Egger’s regression intercept = 16.93, 95% CI = [2.96, 30.90], t = 2.38, *p* = 0.04), and combined depressive and anxiety symptoms (Egger’s regression intercept = 0.59, 95% CI = [0.20, 0.97], *t* = 3.00, *p* = 0.04). Results were consistent after adjusting for the publication bias. Full results of publication bias statistics are summarized in Supplementary Material 8.

### Moderator analysis

The effect sizes between routine disruptions and psychiatric symptoms were significant for physical activity (*r* = 0.06, *p* < 0.01), sleep (*r* = 0.10, *p* = 0.03), unspecified generic routines (*r* = 0.26, *p* < 0.001), and combined multiple routines (*r* = 0.21, *p* < 0.01) and marginally significant for eating (*r* = 0.11, *p* = 0.05) and smoking (*r* = 0.05, *p* = 0.05). Effect sizes were not significant for leisure activities, social activities, work/study, home activities, and alcohol (*p*s ≥ 0.15). The association was comparable between primary and secondary routines (*Q* = 1.03, *p* = 0.31). Routine-symptom associations were significant when daily routine disruptions were defined and assessed as regularity (*r* = 0.22, *p* < 0.001) and change in capability (*r* = 0.14, *p* < 0.01) but not change in frequency (*r* = 0.00, *p* = 0.83). Effect sizes were significant for disruptions to non-internet-based routines (*r* = 0.12, *p* < 0.001; internet-based: *r* =  − 0.05, *p* = 0.09). The associations were independent of whether the measures of daily routines were validated or not (*Q* = 1.02, *p* = 0.31).

While the association remained significant independent of sociodemographics (e.g., gender, education level, marital status, employment status, physical comorbidity), it was stronger in geo-temporal contexts with more COVID-19 monthly deaths (*r* = 0.15, *p* < 0.001) relative to fewer deaths (*r* = 0.06, *p* < 0.001; *Q* = 11.25, *p* < 0.01), and those with lower COVID-19 economic support index (*r* = 0.17, *p* < 0.001) relative to higher index (*r* = 0.06, *p* < 0.01; *Q* = 14.01, *p* < 0.001). Routine disruptions were associated with higher psychiatric symptoms among studies conducted in Asia (*r* = 0.12, *p* < 0.01), Europe (*r* = 0.11, *p* < 0.001), and Oceania (*r* = 0.03, *p* < 0.001), but not in North America (*r* = 0.18, *p* = 0.12) and South America (*r* = -0.01, *p* = 0.81), while the associations were independent of country-level income (*Q* = 2.00, *p* = 0.16). The associations between routine disruptions and outcomes were stronger in prospective studies (*r* = 0.24, *p* < 0.01) than in cross-sectional studies (*r* = 0.10, *p* < 0.001; *Q* = 6.67, *p* < 0.01). Follow-up durations did not moderate the routine-symptom associations (*B* = 0.01, *p* = 0.17). The result showed that the associations were not significantly different between the six studies accounting for over 80% of total respondents and the remaining 47 studies (Q = 0.59, *p* = 0.44). The results of the moderator analyses are summarized in Table 3.

**Table 3:** Moderators of the associations between daily routine disruptions (combined across types) and psychiatric symptoms (53 studies, 51 independent samples)

**Moderator**		**Psychiatric symptoms**		
	***k***	**Statistic type**	**Statistic value [95% CI]**	***p***
***Psychiatric symptoms***				
**Model 1 Psychiatric symptoms**				
*Subgroup differences*	–	*Q*-value	32.10	** < 0.001**
Depressive symptoms	62	Pearson’s *r*	0.13 [0.06; 0.20]	** < 0.001**
Anxiety symptoms	38	Pearson’s *r*	0.12 [0.06; 0.17]	** < 0.001**
Post-traumatic stress disorder (PTSD) symptoms	12	Pearson’s *r*	0.03 [− 0.09; 0.15]	**0﻿.56**
Depressive and anxiety symptoms	6	Pearson’s *r*	0.01 [− 0.01; 0.02]	0.38
General psychological distress^a^	27	Pearson’s *r*	0.09 [0.02; 0.16]	**0.02**
***Daily routine disruptions***				
**Model 2 Category**^**b**^				
*Subgroup differences*	–	*Q*-value	1.03	0.31
Primary routines	31	Pearson’s *r*	0.07 [0.01; 0.14]	**0.02**
Secondary routines	75	Pearson’s *r*	0.04 [0.01; 0.07]	**0.01**
**Model 3 Type**				
*Subgroup differences*	–	*Q*-value	31.76	** < 0.001**
Physical activity	19	Pearson’s *r*	0.06 [0.03; 0.10]	** <0.01**
Eating	7	Pearson’s *r*	0.11 [− 0.00; 0.21]	0.05
Sleep	14	Pearson’s *r*	0.10 [0.01; 0.20]	**0.03**
Social activities	16	Pearson’s *r*	0.04 [− 0.03; 0.10]	0.27
Leisure activities	16	Pearson’s *r*	0.06 [− 0.06; 0.17]	0.29
Work/Study	15	Pearson’s *r*	− 0.03 [− 0.09; 0.04]	**0.41 **
Home activities	10	Pearson’s *r*	0.01 [− 0.13; 0.15]	**0.86**
Smoking	4	Pearson’s *r*	0.05 [0.00; 0.09]	0.05
Alcohol	5	Pearson’s *r*	0.08 [− 0.04; 0.20]	0.15
Combined multiple routines	11	Pearson’s *r*	0.21 [0.10; 0.32]	**<0.01**
Unspecified generic routines	28	Pearson’s *r*	0.26 [0.13; 0.38]	**<0.001**
**Model 4 Definition**				
*Subgroup differences*	–	*Q*-value	38.74	** < 0.001**
Regularity	53	Pearson’s *r*	0.22 [0.15; 0.29]	** < 0.001**
Frequency change	71	Pearson’s *r*	0.00 [− 0.03; 0.03]	0.83
Capability change	21	Pearson’s *r*	0.14 [0.06; 0.22]	** < 0.01**
**Model 5 Internet-related or not**				
*Subgroup differences*	–	*Q*-value	26.04	** < 0.001**
No	131	Pearson’s *r*	0.12 [0.08; 0.16]	** < 0.001**
Yes	14	Pearson’s *r*	− 0.05 [− 0.11; 0.01]	0.09
**Model 6 Assessment method**				
*Subgroup differences*	–	*Q*-value	1.02	0.31
Non-validated scale	99	Pearson’s *r*	0.12 [0.07; 0.17]	** < 0.001**
Validated scale	46	Pearson’s *r*	0.08 [0.05; 0.12]	** < 0.001**
***Population demographics***				
**Model 7 Gender**				
Non-male [range: 42.06–100%]	145	Coefficient	− 0.00 [− 0.00; 0.00]	0.20
**Model 8 Education level**				
Without tertiary education [range 0–100%]	80	Coefficient	0.00 [− 0.00; 0.00]	0.56
**Model 9 Marital status**				
Non-married [range 4.98–98.32%]	53	Coefficient	− 0.00 [− 0.01; 0.00]	0.61
**Model 10 Employment status**				
Non-employed [range 0–93.26%]	71	Coefficient	0.00 [− 0.00; 0.00]	0.44
**Model 11 Physical comorbidity**				
ICD-defined physical comorbidity [range 19.66–100%]	29	Coefficient	− 0.00 [− 0.00; 0.00]	0.50
**Model 12 Country-level income**				
*Subgroup differences*	–	*Q*-value	2.00	0.16
High	121	Pearson’s *r*	0.11 [0.07; 0.16]	** < 0.001**
Middle	24	Pearson’s *r*	0.07 [0.01; 0.12]	**0.02**
***Coronavirus-2019 features***				
**Model 13 Continent**				
*Subgroup differences*	–	*Q*-value	23.67	** < 0.001**
Europe	62	Pearson’s *r*	0.11 [0.06; 0.16]	** < 0.001**
Asia	58	Pearson’s *r*	0.12 [0.05; 0.19]	** < 0.01**
Oceania	12	Pearson’s *r*	0.03 [0.02; 0.04]	** < 0.001**
South America	6	Pearson’s *r*	− 0.01 [− 0.14; 0.11]	0.81
North America	5	Pearson’s *r*	0.18 [− 0.07; 0.41]	0.12
Multiple countries	2	Pearson’s *r*	0.33 [− 0.87; 0.97]	0.23
**Model 14 Number of months since COVID-19 outbreak**				
Number of months since COVID-19 outbreak [range 3–29]	128	Coefficient	− 0.00 [− 0.00; 0.00]	0.86
**Model 15 COVID-19 monthly incidence**^**a**^				
*Subgroup differences*	–	*Q*-value	0.12	0.72
Low	68	Pearson’s *r*	0.11 [0.07; 0.15]	** < 0.001**
High	57	Pearson’s *r*	0.10 [0.05; 0.15]	** < 0.001**
**Model 16 COVID-19 monthly death**^**c**^				
*Subgroup differences*	–	*Q*-value	11.25	** < 0.001**
Low	63	Pearson’s *r*	0.06 [0.03; 0.09]	** < 0.001**
High	62	Pearson’s *r*	0.15 [0.10; 0.20]	** < 0.001**
**Model 17 COVID-19 government response index**^**d**^				
*Subgroup differences*	–	*Q*-value	3.35	0.07
Low	68	Pearson’s *r*	0.13 [0.09; 0.17]	** < 0.001**
High	57	Pearson’s *r*	0.08 [0.04; 0.12]	** < 0.001**
**Model 18 COVID-19 containment and health index**^**d**^				
*Subgroup differences*	–	*Q*-value	0.29	0.59
Low	70	Pearson’s *r*	0.11 [0.07; 0.15]	** < 0.001**
High	55	Pearson’s *r*	0.10 [0.06; 0.14]	** < 0.001**
**Model 19 COVID-19 stringency index**^**d**^				
*Subgroup differences*	–	*Q*-value	1.52	0.22
Low	60	Pearson’s *r*	0.12 [0.08; 0.17]	** < 0.001**
High	65	Pearson’s *r*	0.09 [0.05; 0.13]	** < 0.001**
**Model 20 COVID-19 economic support index**^**d**^				
*Subgroup differences*	–	*Q*-value	14.01	** < 0.001**
Low	53	Pearson’s *r*	0.17 [0.12; 0.21]	** < 0.001**
High	72	Pearson’s *r*	0.06 [0.02; 0.10]	** < 0.01**
***Study features***				
**Model 21 Study quality**				
Study quality [range 9–18]	145	Coefficient	0.01 [− 0.01; 0.03]	0.53
**Model 22 Cross-sectional or prospective design**				
*Subgroup differences*	–	*Q*-value	6.67	** < 0.01**
Cross-sectional	140	Pearson’s *r*	0.10 [0.07; 0.14]	** < 0.001**
Prospective	5	Pearson’s *r*	0.24 [0.10; 0.36]	** < 0.01**
**Model 23 Follow-up duration**				
Follow-up duration after intervention (months) [range 3–12]	4	Coefficient	0.01 [− 0.01; 0.04]	0.17
**Model 24 Sample size**				
*Subgroup differences*	–	*Q*-value	0.59	0.44
Large sample^e^ (*N* = 6)	15	Pearson’s r	0.19 [− 0.07; 0.42]	0.14
Small sample (*N* = 47)	130	Pearson’s r	0.10 [0.07; 0.13]	< 0.001

*k* = Number of effect sizes

^a^“General psychological distress” included distress (e.g., “Kessler Psychological Distress Scale–6 (K6)”) and stress (e.g., “Depression Anxiety Stress Scale-21 (DASS-21) Stress Subscale”)

^b^Primary routines included eating, sleep, and home activities; Secondary routines included physical activity, leisure activities, social activities, and work/studies [16, 20]

^c^The unit is monthly cumulative per million individuals. Information was extracted from official websites. “High” and “Low” categories were generated based on median split

^d^The unit is monthly average score. Information was extracted from Oxford Covid-19 Government Response Tracker (OxCGRT; [19]). “High” and “Low” categories were generated based on median split

^e^Big sample studies refer to the six studies which accounting for over 80% of total respondents, namely Lee & Chu [36], Lee et al. [38], Cho et al. [75], Hampshire et al. [24], Sommerlad et al. [60], and Tondokoro et al. [66]

## Discussion

**Fig. 2:**
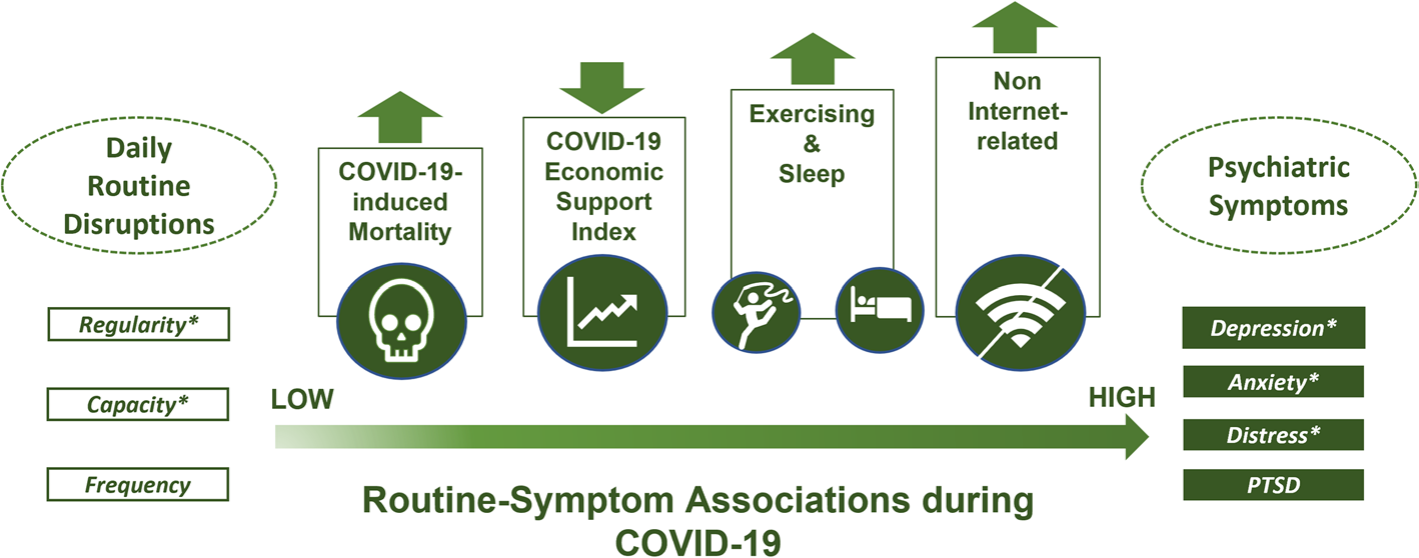
Summary figure of the present findings

This is a systematic review and meta-analysis of 53 studies (51 independent samples) among 910,503 respondents from 32 countries/regions across five different continents, with data collections spanning through the acute phase of COVID-19 to 2.5 years following the outbreak (i.e., mid-2022). We quantitatively synthesized and investigated the moderators of the associations between daily routine disruptions and psychiatric symptoms. On top of establishing the positive pooled associations between disrupted daily routines and psychiatric symptoms, in particular, depressive symptoms, anxiety symptoms, and general psychological distress (*Hypothesis 1*), we further found that the routine-symptom associations differed across continents, monthly cumulative deaths, governmental economic support, study design (i.e., cross-sectional or prospective), and characteristics of routine disruptions (i.e., type, definition, internet-based or not) (*Hypothesis 2*). Summary figure of the present findings is shown in Fig. 2.

### Linking daily routines with psychiatric symptoms

The significant positive associations between disrupted daily routine disruptions and psychiatric symptoms were consistent with existing relevant frameworks, namely the Social Zeitgeber Model [1], the Drive to Thrive (DTT) theory [3, 16], and the Family Routines Framework [2]. The Social Zeitgeber Model [1] theorizes the critical role of social cues in entraining circadian rhythm, with its dysregulation responsible for psychopathology (e.g., bipolar disorders). More specifically, social zeitgebers or time cues derived from the performance of scheduled daily activities (e.g., social contacts, meal/bedtimes, work/studies, leisure activities) serve as referencing anchors for biological rhythms (e.g., sleep–wake cycles). The DTT theory [16] conceptualizes the nature of sustained daily routines by drawing an analogy with “fabrics.” Sustainment of daily routines and the resulting regular daily routines provide a behavioral context that is conducive to psychological resilience in the face of different trauma and chronic stress conditions. The Family Routines Framework [2] suggests that routines performed by the whole family are a unit for adaptive family processes. Family routines refer to certain activities involving two or more family members, which are performed on a day-to-day or week-to-week basis and thus in a repetitive manner with predictable regularity. Family routines can be seen as behavioral patterns of family life [79] that reflect individual family members’ daily routines and associated well-being in an organized and structured manner.

Previous studies have reported maladaptive behavioral consequences of psychiatric disorders [80–82]. Depressive disorders also consist of behavioral manifestations such as reduced physical movement and increased/decreased appetite leading to dietary changes [83]. The DTT theory suggests that daily routines as a behavioral mechanism per se are assessed in terms of regularity and overall structures, whereas the behavioral consequences of psychiatric disorders, i.e., maladaptiveness are defined and assessed as dysfunctions [3, 7]. It is important for future studies to investigate how regularizing daily routines in the aid of mood disorders could reduce maladaptive behavioral byproducts of mood disorders.

We found that disrupted daily routines were selectively associated with higher depressive symptoms, anxiety symptoms, and general psychological distress, but not PTSD symptoms. The results suggested that daily routines could have stronger links to mood disorders than trauma-related disorders. It was argued that the most common consequence of COVID-19 was chronic stress reactions such as depressive and anxiety symptoms and difficulties in adjusting to life stressors, instead of PTSD symptoms that usually arise from life-threatening events [84, 85]. It is important to note that analyses on the former three outcomes were based on more effect sizes; therefore, the subgroup differences could reflect the representativeness of depression, anxiety, and general psychological distress as the most common outcomes among all studies.

### Conceptualizing and assessing daily routines

There has been a deficit of knowledge about the mental health impact of daily activities before COVID-19. One specific routine that has been heavily investigated before COVID-19 was sleep [9, 80, 86]. Regularity in sleep referred to consistent timings marking circadian rhythms, such as overall sleep duration [80], wake time after sleep [86], or perceived regular timings in sleep–wake cycles [9]. Another well-studied routine was physical activity [5, 87]. Most if not all previous studies investigated whether physical activity was done frequently as a healthy lifestyle [5, 87]. These studies could be seen as providing important empirical evidence for the relevance of sleep and physical activity to psychiatric symptoms, but the two daily routines were seldom evaluated in conjunction with other important ones such as chores, leisure, and socializing.

Among the different routines assessed in the current study, we identified associations between disruptions to specific routines and psychiatric symptoms (i.e., physical activity, eating, sleep, smoking, or combined multiple routines/unspecified generic routines). These routines were consistent with those proposed by lifestyle medicine, denoting the evidence-based discipline of applying lifestyle behaviors to the prevention and treatment of medical conditions (e.g., physical exercise, nutrition, sleep health, responsible use of alcohol and substances) [88]. Lifestyle medicine intervention was found to ameliorate symptoms of depression and anxiety [89, 90] and promote health equity among vulnerable populations who are more prone to lifestyle-based chronic diseases [91]. It is also worth noting that symptoms of mental disorders could be predicted by daily routines in aggregate but not in isolation. For example, physical activity could be reduced due to restricted social interactions or the other way round [92], whereas work-from-home could reduce physical activity and healthy eating, and impair sleep quality [15]. The findings were consistent with the theoretical proposition of the adaptive utility of sustaining the structure of daily routines—disruptions to one routine could relate to disruptions to others, whereas sustainment of the regularity of each routine could contribute to an adaptive overall structure that is conducive to stress resilience [1, 7, 79]. The associations with psychiatric symptoms also did not differ between primary routines (i.e., behaviors necessary for maintaining livelihood and biological needs) and secondary routines (i.e., activities reflecting individual circumstances, motivations, and preferences) [7, 93].

The current study, nonetheless, found that disruptions to internet-based daily routines (10% of effect sizes) of leisure and socializing were not associated with symptoms of mental disorders. The eight studies on the disruptions to internet-based routines and psychiatric symptoms investigated online leisure activities (*N* = 12,925), online social activities (*N* = 74,473), and online study (*N* = 397). The non-significant associations were consistent with previous mixed findings on online leisure activities and mental health, with both positive associations with psychological distress and anxiety symptoms [44, 48] and inverse associations with anxiety symptoms, depressive symptoms, PTSD symptoms, and psychological distress [41, 52, 69, 78]. Contrary to the positive associations between disruptions to online social activities and depressive symptoms [60, 78], Gómez-Baya et al. reported inverse associations of the disruptions with anxiety symptoms, depressive symptoms, and PTSD symptoms among pregnant and postpartum women during COVID-19 [78]. In addition, only one study has investigated disruptions to study routine due to restricted internet access among a small student sample (*N* = 397) in a specific sociocultural context of Pakistan [28]. The positive associations between internet-related study routine disruptions and psychiatric symptoms need more solid empirical evidence to support. The inconsistent associations between internet-related routines and psychiatric symptoms actually resembled previous evidence in pre-pandemic data. Screen time was found to have a non-linear dose–response association with depression, with a decreasing risk of depression at less than two hours per day and an increasing risk of depression at more screen time [94]. Another six-wave longitudinal study of the reciprocal relationships between depressive symptoms and screen media use revealed no consistent support for the positive bidirectional associations [95]. Our study supplemented previous evidence by demonstrating internet use is a dynamic phenomenon during large-scale disasters like the COVID-19 pandemic, during which internet replaced some of our usual face-to-face daily routines, such as socializing, leisure, and work. In evaluating the mental health impact of online daily routines, future studies might need to take into account relevant factors such as job-related productivity and satisfaction (i.e., online work)[96], age-related differences (i.e., online socializing and leisure) [97], and generic factors such as digital literacy [98]. Internet behaviors could have been minimally affected by infection control rules [99, 100]. Digital technology has also been suggested to mitigate lockdown emotional consequences such as loneliness [99]. More studies should investigate how the internet might aid everyday adjustment and mental health amid large-scale disasters, such as incorporation of digital elements to facilitate the performance of daily routines and how that in turn brings about positive mental health benefits [101]. Valid and reliable assessment tools of online behaviors pertinent to aspects of daily living other than leisure and socializing are needed. In addition, this line of work should be aware of the disparity of digital literacy that might reduce the benefit of internet-based sustainment of daily routines among individuals who are older, are less educated, and have low income [102, 103].

Beyond routine types, we found a significant moderating effect of the definition and assessment, significant only for regularity and capability but not frequency. The findings suggested that disruptions might be more important to refer to a stable pattern and/or perceptions of being capable of performing well more than frequency. In addition, this meta-analysis only included daily routines that reflected change/non-change since COVID-19—regularity, change in frequency, and change in capability, in order to address the aim of investigating the routine-symptom association amid COVID. The current analysis could preclude preexisting patterns and characteristics of daily routines that might reflect large individual differences not directly related to the impact of COVID-19 [104, 105].

### Population characteristics

The current review of evidence under COVID-19 did not support the moderating role of socioeconomic status in the associations between daily routines and symptoms of mental disorders. One explanation is that studies in the current review did not assess the facets of socioeconomic status relevant to both daily routine disruptions and mental disorders. For example, assets (savings coupled with property ownership) were inversely associated with probable depression among a US population with racial/ethnic disadvantage (Black and Hispanic persons) [106]. Under double stressors of civil unrest and the pandemic, assets could buffer the more vulnerable population (with lower socioeconomic statuses) from the mental health consequences of the stressors [107]. In addition, financial strain was found to relate to higher subsequent depressive and anxiety symptoms through disrupted daily routines [108]. Sleep disturbance due to long working hours was positively associated with depressive symptoms only among those under high (vs. low) financial strain conditions among 792 college students [109]. These findings suggested the importance of considering novel dimensions on the socioeconomic gradient in the routine-symptom associations.

### Spatiotemporal dimensions of COVID-19

The current meta-analysis quantitatively demonstrated that the routine-symptom association was moderated by not only study-wide factors extracted from included studies but also geo-temporal manifestations. COVID-19 and its infection control have been regarded as the unprecedented global contextual factor that impacted daily routines. Only one study has shown a prospective association between improved clarity on daily goals/tasks and decreased depressive symptoms among Wuhan residents in response to the lift of the COVID-19-induced lockdown policies [110]. This current meta-analysis examined different indicators of pandemic severity, including country/region, duration of COVID-19, cumulative incidences, and mortalities, as well as governmental lockdown, health, and economic support responses. We found that the associations between daily routine disruptions and symptoms of mental disorders were stronger in contexts where COVID-19 was more severe, indicated by a higher COVID-19-induced mortality count cumulated over the past month in the specific country/region. The routine-symptom association remained consistent regardless of the strictness in containment (lockdown) or health policies, but the association was stronger with weaker governmental economic support to buffer against the COVID-19 impact. First, routine disruptions in itself were already sufficient to trigger mental health consequences [3, 93], and this could be independent of the extent to which people’s new normal was introduced by external containment (lockdown) and health policies. Second, COVID-19 is a large-scale economic crisis on top of a public health crisis [111], and the adverse mental health impact of routine disruptions could have been exacerbated by secondary economic shock. Taken together with previous findings (e.g., socioeconomic status, assets), this piece of result clarified that the protective importance of socioeconomic resources for mental health during large-scale disasters like COVID-19 could be more on a macro, governmental level instead of the individual level.

## Limitations

This study has some limitations. First, we pooled the effect sizes despite potential conceptual and operational heterogeneity across studies. Second, the number of effect sizes was small for the associations between certain routines and certain mental health outcomes, which could lead to power insufficiency. Third, included studies were biased towards middle-and-high-income countries/regions in Eurasia and therefore other countries/regions could be underrepresented. Particularly, this could have revealed a financial gap in data resources, as low-income countries could have more pressing economic priorities that limit the availability of mental health research, and therefore the specific prevalence and course of mental health conditions in these regions remain marginalized or even absent from the existing literature. In the meantime, however, it is possible that low-income countries experienced more substantial COVID-19 impact given the challenges the disaster posed to their already difficult economic situations. Fourth, there was an imbalance in sample size across studies with six (of all 53) studies accounting for over 80% of total respondents. The routine-symptom associations were significant in the 47 studies with small sample size but not in the six studies with large sample size. The insignificant results could be attributable to non-validated measurements of routines and psychiatric symptoms, but our subgroup analysis ruled out the possibility of measurement error by showing that the associations between routine disruptions and psychiatric symptoms did not differ between validated and non-validated measurements of routines [112]. The result showed that the associations between routine disruptions and psychiatric symptoms were independent of whether the measures were validated or not. Therefore, we have ruled out the possibility of measurement error [112]. Fifth, only four studies were available to show the direction of associations from routines to mental health but not the other way round, although we found that the prospective analyses reported stronger effect size than the cross-sectional analyses. Sixth, due to lockdown and/or social distancing during COVID-19, all included studies were conducted online. The findings may be confounding by the social desirability of self-report studies. Seventh, the results on the associations between routine disruptions and anxiety symptoms, PTSD symptoms, and combined depressive and anxiety symptoms should be interpreted with caution due to significant publication bias, although it has been adjusted for in all analyses of these outcomes.

## Conclusions

Notwithstanding these limitations, the current meta-analysis is one of the most comprehensive and up-to-date systematic synthesis of the association between daily routine disruptions and mental disorders among 910,503 respondents over 32 countries across five continents over 2.5-year period of COVID-19. Such evidence could have potentially important implications for science and practice due to the following reasons. First, because the impact of the pandemic has profoundly permeated people’s day-to-day living all round, the COVID-19 era has directed to a blossom of studies that assessed varying aspects of daily activities. With the growing empirical evidence on daily routines and mood disorders in the COVID-19 pandemic, there is an urgent need to conceptualize daily routines and standardize how they are best assessed and quantified in adaptation to large-scale disasters [107, 113]. The current study could benefit more in-depth investigations on which aspects of daily routines could point to cost-effective assessment and intervention systems for mood disorders. Second, the robustness of the associations between routine disruptions and psychiatric symptoms were further demonstrated by showing their sociodemographic invariance. We also comprehensively clarified the concepts and assessments of daily routines and teased out the type and nature of the disruptions that accounted for symptoms of common mental disorders. Larger societal and community contexts, such as disasters, political violence, social movements, and negative qualities of neighborhoods (i.e., crime, dilapidation, and vagrancy) have been directly and indirectly related to negative everyday experiences and poorer mental health [7, 114–116]. Third, COVID is a global natural experiment of both large-scale economic and public health crises [111, 117]. Larger societal and community contexts, such as disasters, political violence, social movements, and negative qualities of neighborhoods (i.e., crime, dilapidation, and vagrancy) have been directly and indirectly related to negative everyday experiences and poorer mental health [7, 114–116]. The current findings have provided a comprehensive evidence base to guide optimal psychological adjustment amid future large-scale disasters, especially those that could bring prolonged rupture to day-to-day living.

### Supplementary Information

**Additional file 1: Supplementary Material 1.** Detailed search algorithms.

**Additional file 2: Supplementary Material 2.** Coding sheet.

**Additional file 3: Supplementary Material 3.** Effect size conversion formula.

**Additional file 4: Supplementary Material 4.** Critical appraisal in individual studies.

**Additional file 5: Supplementary Material 5.** Descriptive details of individual studies included in the meta-analysis (53 studies, 51 independent samples).

**Additional file 6: Supplementary Material 6.** Forest plot for effect sizes of the association between daily routine disruptions (combined across types) and psychiatric symptoms.

**Additional file 7: Supplementary Material 7.** Funnel plots.

**Additional file 8: Supplementary Material 8.** Publication bias statistics (53 studies, 51 independent samples).

## Data Availability

The datasets used and/or analyzed during the current study available from the corresponding author on reasonable request.

## Abbreviations

DTT: Drive to Thrive theory
OxCGRT: Oxford Covid-19 Government Response Tracker
PRISMA: Preferred Reporting Items for Systematic Review and Meta-Analysis
PTSD: Post-traumatic stress disorder
