# Supplementary material for: Daily routine disruptions and psychiatric symptoms amid COVID-19: a systematic review and meta-analysis of data from 0.9 million individuals in 32 countries
Source: BMC Med. 2024 Feb 2;22:49. doi: 10.1186/s12916-024-03253-x (PMC10835995; doi:10.1186/s12916-024-03253-x)
Supplement: Supplementary file 1 — Additional file 1: Supplementary Material 1. Detailed search algorithms. [file 12916_2024_3253_MOESM1_ESM.docx]

**SUPPLEMENTARY MATERIAL 1** Detailed search algorithms

Three sets of keywords were employed to search for following constructs: *COVID-19*, *mental health*, and *routines*. We use the same keywords chains across the four databases. The three field were combined with AND search command. Detailed keywords were demonstrated below:

| **COVID** [COVID 19 OR COVID-19 OR SARS-CoV-2 OR 2019-nCoV OR n-CoV OR coronavirus* OR novel coronavirus* OR betacov* OR novel betacov* OR betacoronavirus OR novel betacoronavirus* OR corona* OR pandemi* OR new coronavirus* OR new betacov* OR new betacoronavirus* OR Severe acute respiratory syndrome coronavirus 2 OR Coronavirus infect*] |
| --- |
| **Mental health** [substance/drug*/alcohol* abuse/dependence OR schizophrenia* personality* disorder* OR anxiety OR anxiety disorder* OR anxiety symptom* OR anxious feeling* OR anxious mood OR depression OR depressive disorder* OR depressive symptom* OR depressed feeling* OR depressed mood* OR post-traumatic stress* OR post-traumatic stress disorder* OR post-traumatic stress symptom* OR post-traumatic stress response* OR traumatic stress OR traumatic stress disorder* OR traumatic symptom* OR traumatic response* OR psychological distress OR psychological symptom* OR psychological dysfunction* OR emotional distress OR psychiatric symptom* OR psychiatric condition* OR mental health OR NSSI OR suicid* OR self-harm OR self-injur* OR self-mutilation OR overdose OR DSH OR parasuicid* OR mutilation* OR Neurodevelopmental disorders OR Disorders of intellectual development OR Developmental speech OR language disorders OR Autism spectrum disorder OR Developmental learning disorder OR Developmental motor coordination disorder OR Attention deficit hyperactivity disorder OR Stereotyped movement disorder OR Schizophrenia OR psychotic disorder OR Schizoaffective disorder OR Schizotypal disorder OR Delusional disorder OR Symptomatic manifestation OR Catatonia OR Mood disorder OR Bipolar disorder OR Cyclothymic disorder OR Dysthymic disorder OR Panic attack OR Rapid cycling OR fear-related disorder OR Panic disorder OR Agoraphobia or phobia OR Social anxiety disorder OR Separation anxiety disorder OR Selective mutism OR Obsessive-compulsive* OR Body dysmorphic disorder OR Olfactory reference disorder OR Hypochondriasis OR Hoarding disorder OR Body-focused repetitive behaviour disorder OR Prolonged grief disorder OR Adjustment disorder OR Reactive attachment disorder OR Disinhibited social engagement disorder OR Dissociative disorder OR Dissociative neurological symptom disorder OR Dissociative amnesia OR Trance disorder OR Possession trance disorder OR Dissociative identity disorder OR Partial dissociative identity disorder OR Depersonalization-derealization disorder OR Feeding disorder OR eating disorder OR Anorexia Nervosa OR Bulimia Nervosa OR Binge eating disorder OR Avoidant-restrictive food intake disorder OR Pica OR Rumination-regurgitation disorder OR Elimination disorder OR Enuresis OR Encopresis OR Bodily distress disorder OR Body integrity dysphoria OR addictive behaviour OR Impulse control disorder OR Pyromania OR Kleptomania OR Compulsive sexual behaviour disorder OR Intermittent explosive disorder OR Gambling disorder OR Gaming disorder OR Secondary impulse control syndrome OR Disruptive behaviour OR dissocial disorder OR Oppositional defiant disorder OR Conduct-dissocial disorder OR Personality disorder OR Prominent personality trait OR Prominent personality pattern OR Paraphilic disorder OR Exhibitionistic disorder OR Voyeuristic disorder OR Pedophilic disorder OR Coercive sexual sadism disorder OR Frotteuristic disorder OR Factitious disorder OR Neurocognitive disorder OR Delirium OR Mild neurocognitive disorder OR Amnestic disorder OR Dementia OR Sleep-wake disorder OR Insomnia* OR Hypersomnolence disorder OR Narcolepsy OR Idiopathic hypersomnia OR Kleine-Levin syndrome OR Sleep-related breathing disorder OR Central sleep apnoeas OR Obstructive sleep apnoea OR Sleep-related hypoventilation OR Sleep-related hypoxemia OR Sleep-related movement disorder OR Restless legs syndrome OR Periodic limb movement disorder OR Sleep-related leg cramps OR Sleep-related bruxism OR Sleep-related rhythmic movement disorder OR sleep myoclonus OR Parasomnia disorder] |
| **Routine** [Everyday* OR Routine* OR Daily* OR Daily routine* OR Daily life* OR daily stress* OR daily hassle*] |
