# Supplementary material for: Daily routine disruptions and psychiatric symptoms amid COVID-19: a systematic review and meta-analysis of data from 0.9 million individuals in 32 countries
Source: BMC Med. 2024 Feb 2;22:49. doi: 10.1186/s12916-024-03253-x (PMC10835995; doi:10.1186/s12916-024-03253-x)
Supplement: Supplementary file 2 — Additional file 2: Supplementary Material 2. Coding sheet. [file 12916_2024_3253_MOESM2_ESM.docx]

**SUPPLEMENTARY MATERIAL 2** Coding sheet.

| **Coding variable** | **Description** |
| --- | --- |
| Pub_No | The number of independent publications included that stemmed from the same sample |
| Mos_Since_COVID | Based on the methodology information on when the data were collected, the number of months since the COVID outbreak (i.e., 2020) |
|  |  |
| Spec_Population | String variable with words  0 = Not specified |
| Sample_Size | Sample size of the study (calculated based on the sample included in the final analyses)  *Note. If multiple papers reported based on the same sample, sample size from the main paper was counted* |
| Age_Mean | Mean age of sample |
| Age_Range | Age range of sample |
| Age_Group | Descriptive summary of the age group of the sample, including for example, children, adolescents, adults (older adults) |
| NonMale_% | The effective proportion of sample that were not male (i.e., female, other gender)  *Note. When calculating “effective proportion”, missing data were removed from the denominator* |
| NonFemale_% | The effective proportion of sample that were not female (i.e., male, other gender)  *Note. When calculating “effective proportion”, missing data were removed from the denominator* |
| Country | The country or countries where the study was conducted (some studies could be multinational) |
| new_cases_per_million | The cumulative number of new cases (per million individuals) in the studied country over the past month when investigation was conducted  *Note. Data were extracted from official database* |
| new_deaths_per_million | The cumulative number of new deaths (per million individuals) in the studied country over the past month when investigation was conducted  *Note. Data were extracted from official database* |
| Government_Response_Index | Oxford Covid-19 Government Response Tracker (OxCGRT) – Government Response Index (monthly average) |
| Containment_Health_Index | Oxford Covid-19 Government Response Tracker (OxCGRT) – Containment and Health Index (monthly average) |
| Stringency_Index | Oxford Covid-19 Government Response Tracker (OxCGRT) – Stringency Index (monthly average) |
| Economic_Support_Index | Oxford Covid-19 Government Response Tracker (OxCGRT) – Economic Support Index (monthly average) |
| Continent | The continent where the study was conducted (some studies could be multi-continental) |
| Income | Study-level income (based on GNI per capita of World Bank statistics) |
| NonTertiary_% | The effective proportion of sample not having tertiary education or above (i.e., secondary education or below)  *Note. When calculating “effective proportion”, missing data were removed from the denominator* |
| NonMarry_% | The effective proportion of sample that were not married nor in stable relationship  *Note. When calculating “effective proportion”, missing data were removed from the denominator* |
| NonEmploy_% | The effective proportion of sample that were not employed (when we were not sure whether the respondents were employed, and the respondents were not part of the missing data, we excluded these individuals from “employed”)  *Note. When calculating “effective proportion”, missing data were removed from the denominator* |
| PhyDisease_min_% | The minimal proportion of sample that were having a physical disease by WHO standard  *Note. Because studies usually separately reported information for different diagnoses and we did not have sufficient information on comorbidity, when generating this variable, we went for the % for the category with the highest proportion, meaning at least X% of the whole sample should be having a physical disease* |
|  |  |
| Study_Design_Observ_Interv | 1 = Observational  2 = Interventional |
| Study_Design_Cross_Pros | 1 = Cross-sectional  2 = Prospective  *Note. Time schedule was determined based on the routines-symptoms association. (For example, if the study itself was prospective but both routines and symptoms were measured concurrently, this was counted as “cross-sectional”)* |
| Fup_Mos | The number of months for the follow-up (only when the study was prospective) |
| RoB_QR | RoB rating – Quality of reporting (0–7, higher score is better quality) |
| RoB_QD | RoB rating – Study design quality (0–7, higher score is better quality) |
| RoB_PB | RoB rating – Possible introduction of bias (0–6, higher score is better quality) |
| RoB_Total | RoB rating – Total score (0–20, higher score is better quality) |
|  |  |
| Symptom | 1 = Depressive symptoms  2 = Anxiety symptoms  3 = Posttraumatic stress disorder (PTSD) symptoms  4= Depressive symptoms and anxiety symptoms combined  5 = General psychological distress (i.e., Distress, Stress) |
| Symptom_Scale | The name of the instrument for psychiatric symptoms  *For narrative synthesis only* |
| Symptom_Subscale | The name of the instrument (subscale) for psychiatric symptoms  *For narrative synthesis only* |
|  |  |
| Routines | Brief descriptions of the routines  *For narrative synthesis only* |
| Routines_Category | 1 = Primary routines  2 = Secondary routines |
| Routines_Type | 1 = Physical activity  2 = Eating  3 = Sleep  4 = Social activities  5 = Leisure activities  6 = Work/studies  7 = Home activities  8= Smoking  9=Alcohol  10 = Combined multiple routines  11 = Unspecified generic routines |
| Routines_Aspect | 1 = Regularity  2 = Change (increase) in frequency  3 = Change (increase) in capability |
| Routines_Internet | 1 = Not related to internet  2 = Related to internet |
| Routines_Assess_Method | 1 = Non-validated self-developed survey  2 = Validated instrument |
| Routines_Scale | The name of the instrument for routines (validated instruments only)  *For narrative synthesis only* |
|  |  |
| ES_Covar | 1 = No covariates adjusted  2 = Covariates adjusted |
| ES_Type | 1 = Odds ratio  2 = r  3 = β  4 = unstandardized B  5 = Chi-squared |
| ES_Value | Finalized effect size value  *Steps involved:*   1. *Raw effect size was extracted from the original paper* 2. *Effect size was converted to Pearson’s r for comparability (with online calculators)* 3. *Positivity/Negativity of the Pearson’s r value (from Step 2) was checked and adjusted to reflect the associations between routines disruptions (negative valence) and psychiatric symptoms (negative valence) – therefore an effect size consistent with the hypothesis should be a positive value* 4. *When the original paper included multiple levels for the same routine variable, these Pearson’s r’s (from Step 3) were averaged into one Pearson’s r* 5. *When the paper included multiple effect sizes for different routine activities but these routine activities belonged to the same category with all routine features being the same, these effect sizes were averaged into one final Pearson’s r*   *(However, if there were multiple routine activities under the same category but they were having different features, such as one internet-related social activity and one internet-unrelated social activity, then the two effect sizes were kept as separate)* |
| ES_SourceNo | The number of effect sizes averaged from Step 4 to Step 5 |
