# Supplementary material for: Daily routine disruptions and psychiatric symptoms amid COVID-19: a systematic review and meta-analysis of data from 0.9 million individuals in 32 countries
Source: BMC Med. 2024 Feb 2;22:49. doi: 10.1186/s12916-024-03253-x (PMC10835995; doi:10.1186/s12916-024-03253-x)
Supplement: Supplementary file 3 — Additional file 3: Supplementary Material 3. Effect size conversion formula. [file 12916_2024_3253_MOESM3_ESM.docx]

**SUPPLEMENTARY MATERIAL 3** Effect size conversion formula.

Standardized regression coefficients (Eq.(1)), χ2 tests (Eq. (2)), and odd ratios (Eq. (3)) was converted into correlation coefficients *r* using the following equations prior to pooling of effect sizes.

$Eq. (1) r=\beta+0.05\lambda$

$Eq. (2) r=\sqrt{\frac{\chi^{2}}{n}}$

$Eq. (3) r=\frac{Log\left( OR \right)*\left( \frac{\sqrt{3}}{\pi} \right)}{\sqrt{\left( Log\left( OR \right)*\left( \frac{\sqrt{3}}{\pi} \right) \right)^{2}+ \frac{{(n_{1}+n_{2})}^{2}}{n_{1}*n_{2}}}}$

Where n denotes sample size; $\lambda$ =1 if $\beta$ is positive and $\lambda$ =0 if *r* is negative.

Correlation coefficients were transformed into normally distributed Fisher’s Z_r_ in order to adjust for skewed *r* distributions:

${Eq. (5) Z}_{r}=0.5ln\frac{(1+r)}{(1-r)}$

Where Z_r_ is Fisher’s Z transformed correlation, ln represents the natural logarithm and r is the reported Pearson product-moment correlation coefficient. The standard error was then computed according to Eq. (5). The effect sizes were weighted by inverse variance using Eq. (6) and back-transformed into correlation coefficients for presentation using Eq. (7). The standard error for the pooled correlation was then computed by Eq. (8).

Eq. (5) SEZ_r_=$\sqrt{\frac{1}{n-3}}$

Eq. (6) Weight Z_r_= n – 3

$Eq. (7) r=\frac{e^{2Zr}-1}{e^{2Zr}+1}$

Eq. (8) SEZ_r_ = $\left( 1-r^{2} \right)(SEZr)$

Where *e* refers to the base of the natural logarithm.
