# Supplementary material for: Daily routine disruptions and psychiatric symptoms amid COVID-19: a systematic review and meta-analysis of data from 0.9 million individuals in 32 countries
Source: BMC Med. 2024 Feb 2;22:49. doi: 10.1186/s12916-024-03253-x (PMC10835995; doi:10.1186/s12916-024-03253-x)
Supplement: Supplementary file 4 — Additional file 4: Supplementary Material 4. Critical appraisal in individual studies. [file 12916_2024_3253_MOESM4_ESM.docx]

**SUPPLEMENTARY MATERIAL 4** Critical appraisal in individual studies.

|  | **Questions** | | | | | | | | | | | | | | | | | | | | **Scoring** | | | |
| --- | --- | --- | --- | --- | --- | --- | --- | --- | --- | --- | --- | --- | --- | --- | --- | --- | --- | --- | --- | --- | --- | --- | --- | --- |
| **Authors** | **1** | **2** | **3** | **4** | **5** | **6** | **7** | **8** | **9** | **10** | **11** | **12** | **13** | **14** | **15** | **16** | **17** | **18** | **19** | **20** | **QR** | **QD** | **PB** | **Total** |
| Aolymat et al. (2022) | 1 | 1 | 2 | 1 | 1 | 1 | 2 | 1 | 2 | 1 | 1 | 1 | 3 | 2 | 1 | 1 | 1 | 2 | 2 | 1 | 6 | 6 | 2 | 14 |
| Candela et al. (2022) | 2 | 1 | 2 | 1 | 3 | 3 | 2 | 1 | 1 | 1 | 1 | 1 | 2 | 2 | 1 | 1 | 1 | 2 | 3 | 1 | 5 | 4 | 3 | 12 |
| Cho et al. (2022) | 2 | 1 | 2 | 1 | 1 | 1 | 1 | 1 | 2 | 1 | 1 | 1 | 2 | 1 | 1 | 1 | 1 | 1 | 2 | 2 | 6 | 5 | 5 | 16 |
| Czenczek-Lewandowska et al. (2021) | 1 | 1 | 1 | 1 | 1 | 1 | 2 | 1 | 1 | 2 | 1 | 1 | 3 | 2 | 1 | 1 | 1 | 1 | 2 | 1 | 6 | 7 | 3 | 16 |
| Fila-Witecka et al. (2021) | 1 | 1 | 2 | 1 | 3 | 3 | 2 | 1 | 1 | 1 | 1 | 1 | 3 | 2 | 1 | 1 | 1 | 1 | 2 | 1 | 7 | 5 | 2 | 14 |
| Gómez-Baya et al. (2022) | 1 | 1 | 2 | 1 | 1 | 2 | 2 | 1 | 2 | 1 | 1 | 1 | 3 | 2 | 1 | 1 | 1 | 1 | 2 | 1 | 7 | 6 | 1 | 14 |
| Hampshire et al. (2021) | 1 | 1 | 2 | 1 | 1 | 1 | 1 | 1 | 1 | 1 | 1 | 1 | 3 | 1 | 1 | 1 | 1 | 1 | 1 | 1 | 7 | 5 | 4 | 16 |
| Heesen et al. (2022) | 1 | 1 | 1 | 1 | 1 | 2 | 1 | 1 | 1 | 1 | 1 | 1 | 1 | 1 | 1 | 1 | 1 | 1 | 2 | 1 | 7 | 7 | 4 | 18 |
| Ho et al. (2022) | 1 | 1 | 1 | 1 | 1 | 1 | 2 | 1 | 1 | 1 | 1 | 1 | 2 | 2 | 1 | 1 | 1 | 1 | 3 | 1 | 7 | 6 | 4 | 17 |
| Hou et al. (2021) | 1 | 1 | 2 | 1 | 1 | 1 | 2 | 1 | 1 | 1 | 1 | 1 | 3 | 2 | 1 | 1 | 1 | 1 | 2 | 1 | 7 | 6 | 3 | 16 |
| Inam et al. (2022) | 1 | 1 | 1 | 1 | 1 | 2 | 1 | 1 | 2 | 1 | 1 | 1 | 3 | 1 | 1 | 1 | 1 | 1 | 2 | 1 | 7 | 7 | 3 | 17 |
| Jang et al. (2022) | 1 | 1 | 2 | 1 | 1 | 1 | 2 | 1 | 2 | 1 | 1 | 1 | 3 | 2 | 1 | 1 | 1 | 1 | 1 | 1 | 7 | 5 | 2 | 14 |
| Kahawage et al. (2022) | 1 | 1 | 2 | 1 | 1 | 2 | 2 | 1 | 1 | 1 | 1 | 1 | 2 | 2 | 1 | 1 | 1 | 1 | 1 | 1 | 7 | 5 | 3 | 15 |
| Kim et al. (2021) | 1 | 1 | 2 | 1 | 1 | 1 | 2 | 1 | 1 | 1 | 1 | 1 | 3 | 2 | 1 | 1 | 1 | 1 | 2 | 1 | 7 | 6 | 3 | 16 |
| Kornilaki (2022) | 1 | 1 | 2 | 1 | 1 | 2 | 2 | 1 | 2 | 1 | 1 | 1 | 3 | 2 | 1 | 1 | 1 | 1 | 3 | 1 | 7 | 5 | 1 | 13 |
| Kua et al. (2022) | 1 | 1 | 2 | 1 | 1 | 1 | 2 | 1 | 1 | 1 | 1 | 1 | 3 | 2 | 1 | 1 | 1 | 1 | 2 | 1 | 7 | 6 | 3 | 16 |
| Lai et al. (2022) | 1 | 1 | 2 | 1 | 1 | 1 | 1 | 1 | 1 | 1 | 1 | 1 | 2 | 2 | 1 | 1 | 1 | 1 | 2 | 1 | 7 | 6 | 5 | 18 |
| Lee & Chu (2022) | 1 | 1 | 2 | 1 | 1 | 1 | 2 | 1 | 1 | 1 | 1 | 1 | 3 | 2 | 1 | 1 | 1 | 1 | 2 | 1 | 7 | 6 | 3 | 16 |
| Lee et al. (2022) | 1 | 1 | 2 | 1 | 1 | 1 | 2 | 1 | 2 | 1 | 1 | 1 | 2 | 2 | 1 | 1 | 1 | 1 | 2 | 1 | 7 | 6 | 3 | 16 |
| Li, Liang et al. (2022) | 1 | 1 | 2 | 1 | 1 | 1 | 2 | 1 | 1 | 1 | 1 | 1 | 2 | 2 | 1 | 1 | 1 | 1 | 2 | 1 | 7 | 6 | 4 | 17 |
| *Tao, et al. (2022)* | *1* | *1* | *2* | *1* | *1* | *1* | *2* | *1* | *1* | *1* | *1* | *1* | *3* | *2* | *1* | *1* | *1* | *1* | *2* | *1* | *7* | *6* | *3* | *16* |
| Li, Liu et al. (2022) | 1 | 1 | 2 | 1 | 1 | 2 | 2 | 1 | 2 | 1 | 1 | 1 | 3 | 2 | 1 | 1 | 1 | 1 | 2 | 1 | 7 | 6 | 1 | 14 |
| Lin et al. (2023) | 1 | 1 | 2 | 1 | 1 | 2 | 2 | 1 | 1 | 1 | 2 | 1 | 1 | 2 | 1 | 1 | 1 | 1 | 2 | 1 | 6 | 6 | 2 | 14 |
| Liu et al. (2021) | 1 | 1 | 2 | 1 | 2 | 2 | 2 | 1 | 1 | 1 | 1 | 1 | 3 | 2 | 1 | 1 | 1 | 1 | 1 | 1 | 7 | 4 | 2 | 13 |
| *Ren et al. (2021)* | *1* | *1* | *2* | *1* | *1* | *1* | *2* | *1* | *2* | *1* | *1* | *1* | *2* | *2* | *1* | *1* | *1* | *1* | *2* | *1* | *7* | *6* | *3* | *16* |
| *Yuan et al. (2022)* | *1* | *1* | *2* | *1* | *1* | *2* | *2* | *1* | *2* | *1* | *1* | *1* | *3* | *2* | *1* | *1* | *1* | *1* | *2* | *1* | *7* | *6* | *1* | *14* |
| Lotzin et al. (2022) | 1 | 1 | 1 | 1 | 1 | 2 | 2 | 1 | 2 | 1 | 1 | 1 | 3 | 2 | 1 | 1 | 1 | 1 | 2 | 1 | 7 | 7 | 1 | 15 |
| Martinelli et al. (2021) | 1 | 1 | 2 | 2 | 1 | 2 | 2 | 1 | 2 | 1 | 1 | 1 | 3 | 2 | 1 | 1 | 1 | 1 | 2 | 1 | 6 | 6 | 1 | 13 |
| Matsuo et al. (2022) | 1 | 1 | 2 | 1 | 2 | 2 | 2 | 1 | 2 | 1 | 1 | 1 | 3 | 2 | 1 | 1 | 1 | 1 | 2 | 1 | 7 | 5 | 1 | 13 |
| McGoron et al. (2022) | 1 | 1 | 2 | 1 | 1 | 3 | 1 | 1 | 1 | 1 | 1 | 1 | 2 | 2 | 1 | 1 | 1 | 1 | 1 | 1 | 7 | 5 | 4 | 16 |
| McMahon et al. (2022) | 1 | 1 | 2 | 2 | 2 | 2 | 2 | 1 | 1 | 1 | 1 | 1 | 3 | 2 | 1 | 1 | 1 | 1 | 2 | 1 | 6 | 5 | 2 | 13 |
| Nyberg et al. (2023) | 1 | 1 | 2 | 1 | 1 | 2 | 2 | 1 | 2 | 1 | 1 | 1 | 1 | 1 | 1 | 1 | 1 | 1 | 2 | 1 | 7 | 6 | 2 | 15 |
| Peñaranda et al. (2022) | 1 | 1 | 1 | 1 | 1 | 2 | 2 | 1 | 2 | 1 | 1 | 1 | 3 | 2 | 1 | 1 | 1 | 1 | 2 | 1 | 7 | 7 | 1 | 15 |
| Pensgaard et al. (2021) | 1 | 1 | 2 | 1 | 1 | 1 | 2 | 1 | 1 | 1 | 1 | 1 | 1 | 2 | 1 | 1 | 1 | 1 | 1 | 1 | 7 | 5 | 3 | 15 |
| Rens et al. (2021) | 1 | 1 | 2 | 1 | 1 | 1 | 2 | 1 | 2 | 1 | 1 | 1 | 3 | 2 | 1 | 1 | 1 | 1 | 2 | 1 | 7 | 6 | 2 | 15 |
| Ryu et al. (2021) | 1 | 1 | 2 | 1 | 1 | 3 | 2 | 1 | 1 | 1 | 1 | 1 | 3 | 2 | 1 | 1 | 1 | 1 | 1 | 1 | 7 | 5 | 2 | 14 |
| Sato et al. (2021) | 1 | 1 | 2 | 1 | 1 | 1 | 2 | 1 | 1 | 1 | 1 | 1 | 3 | 2 | 1 | 1 | 1 | 1 | 2 | 1 | 7 | 6 | 3 | 16 |
| Schneider et al. (2023) | 1 | 1 | 3 | 1 | 1 | 1 | 2 | 1 | 2 | 1 | 1 | 1 | 3 | 2 | 1 | 1 | 1 | 1 | 2 | 1 | 7 | 6 | 2 | 15 |
| Şentürk et al. (2021) | 1 | 1 | 2 | 1 | 2 | 2 | 2 | 1 | 2 | 1 | 1 | 1 | 3 | 2 | 1 | 1 | 1 | 1 | 2 | 1 | 7 | 5 | 1 | 13 |
| Shatla et al. (2020) | 1 | 2 | 2 | 2 | 2 | 2 | 2 | 2 | 2 | 1 | 1 | 1 | 3 | 2 | 1 | 1 | 1 | 1 | 2 | 1 | 6 | 3 | 1 | 10 |
| Shoshaniet al. (2022) | 1 | 1 | 2 | 1 | 1 | 1 | 2 | 1 | 1 | 1 | 1 | 1 | 3 | 2 | 1 | 1 | 1 | 1 | 3 | 1 | 7 | 5 | 3 | 15 |
| Sommerlad et al. (2021) | 1 | 1 | 2 | 1 | 1 | 2 | 2 | 1 | 2 | 1 | 1 | 1 | 3 | 2 | 1 | 1 | 1 | 1 | 2 | 1 | 7 | 6 | 1 | 14 |
| Stanton et al. (2020) | 1 | 1 | 2 | 1 | 2 | 2 | 2 | 1 | 2 | 1 | 1 | 1 | 3 | 2 | 1 | 1 | 1 | 1 | 2 | 1 | 7 | 5 | 1 | 13 |
| Sum et al. (2023) | 1 | 1 | 2 | 1 | 2 | 2 | 2 | 1 | 2 | 1 | 1 | 1 | 2 | 2 | 1 | 1 | 1 | 1 | 1 | 1 | 7 | 4 | 2 | 13 |
| Tanaka et al. (2022) | 1 | 1 | 1 | 1 | 1 | 2 | 1 | 1 | 1 | 1 | 1 | 1 | 2 | 1 | 1 | 1 | 1 | 1 | 1 | 1 | 7 | 6 | 5 | 18 |
| Tanikaga et al. (2023) | 1 | 1 | 2 | 1 | 1 | 2 | 2 | 1 | 1 | 1 | 1 | 1 | 2 | 2 | 1 | 1 | 1 | 1 | 2 | 1 | 7 | 6 | 3 | 16 |
| Tondokoro et al. (2023) | 1 | 1 | 2 | 1 | 1 | 1 | 1 | 1 | 2 | 1 | 1 | 1 | 3 | 1 | 1 | 1 | 1 | 1 | 2 | 1 | 7 | 6 | 4 | 17 |
| Valdés et al. (2022) | 1 | 1 | 2 | 1 | 1 | 2 | 2 | 1 | 1 | 1 | 1 | 1 | 3 | 2 | 1 | 1 | 1 | 1 | 1 | 1 | 7 | 5 | 2 | 14 |
| Vrublevska, et al. (2022) | 1 | 1 | 2 | 1 | 1 | 1 | 2 | 1 | 2 | 1 | 1 | 1 | 3 | 2 | 1 | 1 | 1 | 1 | 1 | 1 | 7 | 5 | 2 | 14 |
| Wang et al. (2022) | 1 | 1 | 2 | 1 | 1 | 2 | 2 | 1 | 2 | 1 | 1 | 1 | 3 | 2 | 1 | 1 | 1 | 1 | 2 | 1 | 7 | 6 | 1 | 14 |
| Waston et al. (2023) | 1 | 1 | 2 | 1 | 2 | 2 | 2 | 1 | 1 | 1 | 1 | 1 | 2 | 1 | 1 | 1 | 1 | 1 | 2 | 1 | 7 | 5 | 4 | 16 |
| Wu et al. (2023) | 2 | 1 | 2 | 1 | 2 | 2 | 2 | 1 | 2 | 1 | 2 | 1 | 3 | 2 | 1 | 1 | 1 | 2 | 2 | 3 | 4 | 4 | 1 | 9 |
| Yılmaz & Önal. (2023) | 1 | 1 | 1 | 1 | 1 | 2 | 2 | 1 | 1 | 1 | 1 | 1 | 3 | 2 | 1 | 1 | 1 | 1 | 2 | 1 | 7 | 7 | 2 | 16 |

Note. 1 = Yes, 2 = No, 3 = Do not know. Items 13 and 19 are reverse-scored when generating subtotal and total scores. Items in italic (i.e., “Tao et al. (2022)”, “Ren et al. (2021)”, “Yuan et al. (2022)”) are independent papers but with overlapping samples.

Abbreviations. QR = Quality of reporting (including items 1, 4, 10, 11, 12, 16, 18, with light yellow shade); QD = Study design quality (including items 2, 3, 5, 8, 17, 19, 20, with light green shade); PB = Possible introduction of biases in the study (including items 6, 7, 9, 13, 14, 15, with white shade).
