# Supplementary material for: Daily routine disruptions and psychiatric symptoms amid COVID-19: a systematic review and meta-analysis of data from 0.9 million individuals in 32 countries
Source: BMC Med. 2024 Feb 2;22:49. doi: 10.1186/s12916-024-03253-x (PMC10835995; doi:10.1186/s12916-024-03253-x)
Supplement: Supplementary file 5 — Additional file 5: Supplementary Material 5. Descriptive details of individual studies included in the meta-analysis (53 studies, 51 independent samples). [file 12916_2024_3253_MOESM5_ESM.docx]

**SUPPLEMENTARY MATERIAL 5** Descriptive details of individual studies included in the meta-analysis (53 studies, 51 independent samples).

| **References** | **Study quality** | **Study design (*observational, interventional*)** | **Study design (*cross-sectional, prospective*)** | **Follow-up duration, *in months*** | **Country** | **Months since COVID** | **COVID severity, *incidence*** | **COVID severity, *death*** | **COVID policy, *government response index*** | **COVID policy, *containment and health index*** | **COVID policy, *stringency index*** | **COVID policy, *economic support index*** | **Sample size** | **Age, *mean*** | **Age, *range*** | **Non-male *(%)*** | **Without tertiary education *(%)*** | **Non-married *(%)*** | **Non-employed *(%)*** | **Physical comorbidity *(%)*** | **Country-level income** | **Psychiatric symptoms** | **Routines, *category*** | **Routines, *type*** | **Routines, *aspect*** | **Routines, *internet relation*** | **Routines, *assessment method*** |
| --- | --- | --- | --- | --- | --- | --- | --- | --- | --- | --- | --- | --- | --- | --- | --- | --- | --- | --- | --- | --- | --- | --- | --- | --- | --- | --- | --- |
| Aolymat et al. (2022) | 14 | Observational | Cross-sectional | N/A | Jordan | 10 | 5250.546 | 63.350 | 64.279 | 68.106 | 79.449 | 37.500 | 385 | 19.89 | No report | 100.00 | No report | No report | No report | No report | Middle | Depressive symptoms | N/A | Unspecified generic routines | Regularity | No | Non-validated survey |
|  |  |  |  |  |  |  |  |  |  |  |  |  |  |  |  |  |  |  |  |  |  | Anxiety symptoms | N/A | Unspecified generic routines | Regularity | No | Non-validated survey |
|  |  |  |  |  |  |  |  |  |  |  |  |  |  |  |  |  |  |  |  |  |  | General psychological distress | N/A | Unspecified generic routines | Regularity | No | Non-validated survey |
| Candela et al. (2022) | 12 | Observational | Prospective | No report | Spain | 5 | 486.558 | 72.015 | 66.735 | 64.170 | 78.584 | 84.677 | 40 | 61.00 | 39, 86 | 90.00 | No report | No report | No report | 100.00 | High | Depressive and anxiety symptoms combined | Primary | Sleep | Change in capability | No | Validated instrument |
| Cho et al. (2022) | 16 | Observational | Cross-sectional | N/A | South Korea | 10 | 52.088 | 0.984 | 57.342 | 58.391 | 57.645 | 50.000 | 223,031 | 48.80 | No report | 54.39 | 65.87 | 37.00 | 42.88 | No report | High | Depressive symptoms | Secondary | Physical activity | Change in frequency | No | Non-validated survey |
|  |  |  |  |  |  |  |  |  |  |  |  |  |  |  |  |  |  |  |  |  |  |  | Secondary | Social activities | Change in frequency | No | Non-validated survey |
|  |  |  |  |  |  |  |  |  |  |  |  |  |  |  |  |  |  |  |  |  |  |  | Secondary | Alcohol | Change in frequency | No | Non-validated survey |
| Czenczek-Lewandowska et al. (2021) | 16 | Observational | Cross-sectional | N/A | Poland | 4 | 265.574 | 14.829 | 61.683 | 65.138 | 84.197 | 37.500 | 506 | 24.67 | 18, 34 | 70.16 | 53.95 | No report | 67.00 | No report | High | Anxiety symptoms | Secondary | Physical activity | Change in frequency | No | Validated instrument |
|  |  |  |  |  |  |  |  |  |  |  |  |  |  |  |  |  |  |  |  |  |  |  | Primary | Sleep | Change in capability | No | Validated instrument |
| Fila-Witecka et al. (2021) | 14 | Observational | Cross-sectional | N/A | Poland | 6 | 265.523 | 9.635 | 49.618 | 51.346 | 57.778 | 37.500 | 980 | 22.24 | No report | 74.80 | 0.00 | No report | 74.29 | No report | High | General psychological distress | Secondary | Physical activity | Change in frequency | No | "Yes/No" |
|  |  |  |  |  |  |  |  |  |  |  |  |  |  |  |  |  |  |  |  |  |  |  | Primary | Eating | Regularity | No | "Yes/No" |
|  |  |  |  |  |  |  |  |  |  |  |  |  |  |  |  |  |  |  |  |  |  |  | Secondary | Leisure activities | Regularity | No | "Yes/No" |
|  |  |  |  |  |  |  |  |  |  |  |  |  |  |  |  |  |  |  |  |  |  |  | Secondary | Social activities | Regularity | No | "Yes/No" |
|  |  |  |  |  |  |  |  |  |  |  |  |  |  |  |  |  |  |  |  |  |  |  | Primary | Home activities | Regularity | No | "Yes/No" |
|  |  |  |  |  |  |  |  |  |  |  |  |  |  |  |  |  |  |  |  |  |  |  | N/A | Unspecified generic routines | Regularity | No | "Yes/No" |
|  |  |  |  |  |  |  |  |  |  |  |  |  |  |  |  |  |  |  |  |  |  | PTSD symptoms | Secondary | Physical activity | Change in frequency | No | "Yes/No" |
|  |  |  |  |  |  |  |  |  |  |  |  |  |  |  |  |  |  |  |  |  |  |  | Primary | Eating | Regularity | No | "Yes/No" |
|  |  |  |  |  |  |  |  |  |  |  |  |  |  |  |  |  |  |  |  |  |  |  | Secondary | Leisure activities | Regularity | No | "Yes/No" |
|  |  |  |  |  |  |  |  |  |  |  |  |  |  |  |  |  |  |  |  |  |  |  | Secondary | Social activities | Regularity | No | "Yes/No" |
|  |  |  |  |  |  |  |  |  |  |  |  |  |  |  |  |  |  |  |  |  |  |  | Primary | Home activities | Regularity | No | "Yes/No" |
|  |  |  |  |  |  |  |  |  |  |  |  |  |  |  |  |  |  |  |  |  |  |  | N/A | Unspecified generic routines | Regularity | No | "Yes/No" |
| Gómez-Baya et al. (2022) | 14 | Observational | Cross-sectional | N/A | Spain | No report | No report | No report | No report | No report | No report | No report | 3,356 | 33.67 | No report | 100.00 | No report | No report | No report | No report | High | Anxiety symptoms | Secondary | Leisure activities | Change in frequency | Yes | Validated instrument |
|  |  |  |  |  |  |  |  |  |  |  |  |  |  |  |  |  |  |  |  |  |  |  |  |  |  | No |  |
|  |  |  |  |  |  |  |  |  |  |  |  |  |  |  |  |  |  |  |  |  |  |  | Secondary | Social activities | Change in frequency | Yes | Validated instrument |
|  |  |  |  |  |  |  |  |  |  |  |  |  |  |  |  |  |  |  |  |  |  | Depressive symptoms | Secondary | Leisure activities | Change in frequency | Yes | Validated instrument |
|  |  |  |  |  |  |  |  |  |  |  |  |  |  |  |  |  |  |  |  |  |  |  |  |  |  | No |  |
|  |  |  |  |  |  |  |  |  |  |  |  |  |  |  |  |  |  |  |  |  |  |  | Secondary | Social activities | Change in frequency | Yes | Validated instrument |
|  |  |  |  |  |  |  |  |  |  |  |  |  |  |  |  |  |  |  |  |  |  | PTSD symptoms | Secondary | Leisure activities | Change in frequency | Yes | Validated instrument |
|  |  |  |  |  |  |  |  |  |  |  |  |  |  |  |  |  |  |  |  |  |  |  |  |  |  | No |  |
|  |  |  |  |  |  |  |  |  |  |  |  |  |  |  |  |  |  |  |  |  |  |  | Secondary | Social activities | Change in frequency | Yes | Validated instrument |
|  |  |  |  |  |  |  |  |  |  |  |  |  |  |  |  |  |  |  |  |  |  |  |  |  |  | No |  |
| Hampshire et al. (2021) | 16 | Observational | Cross-sectional | N/A | United Kingdom | 6 | 413.592 | 58.867 | 70.275 | 66.029 | 70.866 | 100.000 | 379,875 | 44.77 | No report | 46.51 | 36.55 | No report | 30.98 | No report | High | Depressive symptoms | N/A | Unspecified generic routines | Regularity | No | Non-validated survey |
|  |  |  |  |  |  |  |  |  |  |  |  |  |  |  |  |  |  |  |  |  |  | Anxiety symptoms | N/A | Unspecified generic routines | Regularity | No | Non-validated survey |
| Heesen et al. (2022) | 18 | Observational | Prospective | 6 | Germany | 25 | 33499.738 | 45.301 | 55.362 | 57.050 | 41.493 | 43.548 | 98 | 52.10 | No report | 70.40 | 100.00 | No report | No report | 38.89 | High | Depressive and anxiety symptoms combined | Secondary | Social activities | Change in frequency | Yes | Validated instrument |
| Ho et al. (2022) | 17 | Observational | Cross-sectional | N/A | Hong Kong SAR | 4 | 47.176 | 0.000 | 65.542 | 60.615 | 66.207 | 100.000 | 303 | No report | 20, 64 | 76.57 | 0.00 | No report | 0.00 | No report | High | Depressive symptoms | Secondary | Work/studies | Change in capability | No | Validated instrument |
|  |  |  |  |  |  |  |  |  |  |  |  |  |  |  |  |  |  |  |  |  |  | Anxiety symptoms | Secondary | Work/studies | Change in capability | No | Validated instrument |
| Hou et al. (2021) | 16 | Observational | Cross-sectional | N/A | Hong Kong SAR | 3 | 82.877 | 0.267 | 55.446 | 49.079 | 51.342 | 100.000 | 4,021 | 46.10 | 15, 92 | 55.21 | 52.97 | 42.98 | 43.50 | No report | High | Anxiety symptoms | N/A | Combined multiple routines | Regularity | No | Validated instrument |
| Inam et al. (2022) | 17 | Observational | Cross-sectional | N/A | Pakistan | 13 | 279.758 | 6.684 | 65.902 | 64.602 | 61.603 | 75.000 | 397 | 19.90 | 17, 21 | 81.86 | 0.00 | No report | No report | No report | Middle | Depressive and anxiety symptoms combined | Secondary | Social activities | Change in frequency | No | Non-validated survey |
|  |  |  |  |  |  |  |  |  |  |  |  |  |  |  |  |  |  |  |  |  |  |  | Secondary | Work/studies | Change in frequency | Yes | Non-validated survey |
|  |  |  |  |  |  |  |  |  |  |  |  |  |  |  |  |  |  |  |  |  |  |  |  |  |  | No |  |
| Jang et al. (2022) | 14 | Observational | Cross-sectional | N/A | South Korea | 10 | 52.088 | 0.984 | 57.342 | 58.391 | 57.645 | 50.000 | 1,071 | 40.01 | No report | 46.60 | 16.13 | 46.03 | 37.35 | No report | High | Depressive symptoms | N/A | Unspecified generic routines | Regularity | No | Non-validated survey |
| Kahawage et al. (2022) | 15 | Observational | Cross-sectional | N/A | Australia, New Zealand, United Kingdom, US, Canada, and the Netherlands | 9 | No report | No report | No report | No report | No report | No report | 997 | 39.75 | No report | 84.11 | No report | 51.65 | 49.95 | No report | High | Depressive symptoms | N/A | Combined multiple routines | Regularity | No | Validated instrument |
|  |  |  | Prospective | 3 |  |  |  |  |  |  |  |  |  |  |  |  |  |  |  |  |  |  |  |  |  |  |  |
| Kim et al. (2021) | 16 | Observational | Cross-sectional | N/A | South Korea | 5 | 13.569 | 0.444 | 49.615 | 49.558 | 43.563 | 50.000 | 1,492 | 40.30 | 19, 65 | 50.13 | 17.09 | 50.60 | No report | 26.68 | Middle | Depressive symptoms | N/A | Combined multiple routines | Regularity | No | Non-validated survey |
| Kornilaki (2022) | 13 | Observational | Cross-sectional | N/A | Greece | 4 | 131.344 | 9.245 | 61.720 | 63.393 | 84.260 | 50.000 | 1,018 | 21.50 | No report | 83.50 | No report | No report | No report | No report | High | Depressive symptoms | N/A | Unspecified generic routines | Regularity | No | Non-validated survey |
|  |  |  |  |  |  |  |  |  |  |  |  |  |  |  |  |  |  |  |  |  |  | Anxiety symptoms | N/A | Unspecified generic routines | Regularity | No | Non-validated survey |
|  |  |  |  |  |  |  |  |  |  |  |  |  |  |  |  |  |  |  |  |  |  | General psychological distress | N/A | Unspecified generic routines | Regularity | No | Non-validated survey |
| Kua et al. (2022) | 16 | Observational | Cross-sectional | N/A | Singapore | 6 | 1648.919 | 0.531 | 71.491 | 67.419 | 65.619 | 100.000 | 707 | 37.43 | 17, 78 | 83.17 | 21.09 | 44.70 | 0.00 | No report | High | Depressive symptoms | Secondary | Physical activity | Change in frequency | No | Validated instrument |
|  |  |  |  |  |  |  |  |  |  |  |  |  |  |  |  |  |  |  |  |  |  | Anxiety symptoms | Secondary | Physical activity | Change in frequency | No | Validated instrument |
|  |  |  |  |  |  |  |  |  |  |  |  |  |  |  |  |  |  |  |  |  |  | General psychological distress | Secondary | Physical activity | Change in frequency | No | Validated instrument |
|  |  |  |  |  |  |  |  |  |  |  |  |  |  |  |  |  |  |  |  |  |  | PTSD symptoms | Secondary | Physical activity | Change in frequency | No | Validated instrument |
| Lai et al. (2022) | 18 | Observational | Cross-sectional | N/A | Hong Kong SAR | 8 | 205.186 | 4.278 | 73.035 | 69.179 | 66.670 | 100.000 | 2,034 | 43.50 | No report | 54.38 | 48.38 | 46.07 | 41.00 | No report | High | Depressive symptoms | N/A | Combined multiple routines | Regularity | No | Validated instrument |
| Lee & Chu (2022) | 16 | Observational | Cross-sectional | N/A | South Korea | No report | No report | No report | No report | No report | No report | No report | 72,335 | 73.79 | No report | 58.27 | 0.75 | No report | 60.86 | 53.90 | High | Depressive symptoms | Secondary | Physical activity | Change in frequency | No | Non-validated survey |
|  |  |  |  |  |  |  |  |  |  |  |  |  |  |  |  |  |  |  |  |  |  |  | Primary | Sleep | Change in frequency | No | Non-validated survey |
|  |  |  |  |  |  |  |  |  |  |  |  |  |  |  |  |  |  |  |  |  |  |  | Secondary | Social activities | Change in frequency | No | Non-validated survey |
|  |  |  |  |  |  |  |  |  |  |  |  |  |  |  |  |  |  |  |  |  |  |  | Secondary | Alcohol | Change in frequency | No | Non-validated survey |
|  |  |  |  |  |  |  |  |  |  |  |  |  |  |  |  |  |  |  |  |  |  |  | Secondary | Smoking | Change in frequency | No | Non-validated survey |
| Lee et al. (2022) | 16 | Observational | Cross-sectional | N/A | South Korea | No report | No report | No report | No report | No report | No report | No report | 68,207 | 43.80 | 19, 65 | 55.34 | 33.61 | 35.91 | 0.00 | No report | High | Depressive symptoms | N/A | Unspecified generic routines | Regularity | No | Non-validated survey |
| Li, Liang et al. (2022);  Tao, et al. (2022) | 17 | Observational | Prospective | 12 | Hong Kong SAR | 20 | 17.267 | 0.000 | 73.255 | 73.003 | 63.558 | 75.000 | 1,318 | No report | No report | 48.25 | 41.73 | 50.76 | 33.31 | No report | High | Depressive symptoms | N/A | Combined multiple routines | Regularity | No | Validated instrument |
|  |  |  |  |  |  |  |  |  |  |  |  |  |  |  |  |  |  |  |  |  |  | Anxiety symptoms | N/A | Combined multiple routines | Regularity | No | Validated instrument |
| Li, Liu et al. (2022) | 14 | Observational | Cross-sectional | N/A | China | 4 | 1.282 | 0.932 | 54.707 | 61.332 | 60.213 | 8.333 | 1,250 | 39.40 | 18, 72 | 63.68 | 47.12 | No report | 82.88 | No report | Middle | Depressive symptoms | Primary | Sleep | Change in capability | No | Non-validated survey |
| Lin et al. (2023) | 14 | Observational | Cross-sectional | N/A | US | No report | No report | No report | No report | No report | No report | No report | 736 | 25.40 | No report | 78.26 | 0.00 | No report | No report | No report | High | Depressive and anxiety symptoms combined | Primary | Eating | Regularity | No | Non-validated survey |
| Liu et al. (2021);  Ren et al. (2021);  Yuan et al. (2023) | 13 | Observational | Cross-sectional | N/A | China | 4 | 1.282 | 0.932 | 54.707 | 61.332 | 60.213 | 8.333 | 1,594 | 13.13 | No report | 50.50 | 100.00 | No report | No report | No report | Middle | Depressive symptoms | Secondary | Physical activity | Regularity | No | Non-validated survey |
|  |  |  |  |  |  |  |  |  |  |  |  |  |  |  |  |  |  |  |  |  |  |  | Secondary | Leisure activities | Regularity | Yes | Non-validated survey |
|  |  |  |  |  |  |  |  |  |  |  |  |  |  |  |  |  |  |  |  |  |  |  | Secondary | Work/studies | Regularity | No | Validated instrument |
|  |  |  |  |  |  |  |  |  |  |  |  |  |  |  |  |  |  |  |  |  |  |  | Primary | Home activities | Regularity | No | Validated instrument |
|  |  |  |  |  |  |  |  |  |  |  |  |  |  |  |  |  |  |  |  |  |  |  | N/A | Unspecified generic routines | Regularity | No | Validated instrument |
| Lotzin et al. (2022) | 15 | Observational | Cross-sectional | N/A | Austria, Croatia, Georgia, Germany, Greece, Italy, Lithuania, the Netherlands, Poland, Portugal, Sweden | 11 | No report | No report | No report | No report | No report | No report | 4,607 | 43.77 | 18, 89 | 73.56 | No report | No report | 22.01 | No report | High (mostly) and Middle | PTSD symptoms | Primary | Home activities | Change in frequency | No | Non-validated survey |
| Martinelli et al. (2021) Study 1 | 13 | Observational | Cross-sectional | N/A | France | 4 | 1285.675 | 325.516 | 75.000 | 71.430 | 87.960 | 100.000 | 1,332 | 61.05 | No report | 75.98 | No report | 37.09 | No report | No report | High | Anxiety symptoms | Primary | Sleep | Regularity | No | "Yes/No" |
|  |  |  |  |  |  |  |  |  |  |  |  |  |  |  |  |  |  |  |  |  |  |  | N/A | Unspecified generic routines | Regularity | No | Non-validated survey |
| Martinelli et al. (2021) Study 2 | 13 | Observational | Cross-sectional | N/A | France | 4 | 1285.675 | 325.516 | 75.000 | 71.430 | 87.960 | 100.000 | 1,116 | 45.76 | No report | 50.63 | No report | 27.87 | No report | No report | High | Anxiety symptoms | Primary | Sleep | Regularity | No | "Yes/No" |
|  |  |  |  |  |  |  |  |  |  |  |  |  |  |  |  |  |  |  |  |  |  |  | N/A | Unspecified generic routines | Regularity | No | Non-validated survey |
| Matsuo et al. (2022) | 13 | Observational | Cross-sectional | N/A | Japan | 25 | 8043.780 | 3.218 | 59.038 | 53.187 | 47.220 | 100.000 | 166 | No report | No report | 42.17 | 0.00 | No report | 22.89 | No report | High | General psychological distress | Secondary | Social activities | Change in capability | No | Non-validated survey |
|  |  |  |  |  |  |  |  |  |  |  |  |  |  |  |  |  |  |  |  |  |  |  | Secondary | Leisure activities | Change in frequency | Yes | Non-validated survey |
|  |  |  |  |  |  |  |  |  |  |  |  |  |  |  |  |  |  |  |  |  |  |  | Secondary | Work/studies | Change in capability | No | Non-validated survey |
| McGoron et al. (2022) | 16 | Observational | Cross-sectional | N/A | US | 6 | 2427.477 | 67.770 | 63.956 | 64.166 | 70.711 | 62.500 | 152 | No report | No report | 95.27 | 73.57 | 60.54 | No report | No report | High | Depressive symptoms | N/A | Combined multiple routines | Regularity | No | Non-validated survey |
|  |  |  |  |  |  |  |  |  |  |  |  |  |  |  |  |  |  |  |  |  |  | Anxiety symptoms | N/A | Combined multiple routines | Regularity | No | Non-validated survey |
| McMahon et al. (2022) | 13 | Observational | Cross-sectional | N/A | Ireland | 5 | 995.200 | 84.407 | 73.117 | 69.280 | 87.394 | 100.000 | 496 | 28.73 | 18, 73 | 75.20 | No report | No report | No report | No report | High | Depressive symptoms | N/A | Unspecified generic routines | Regularity | No | Non-validated survey |
| Nyberg et al. (2023) | 15 | Observational | Cross-sectional | N/A | Sweden | 18 | 1802.671 | 8.534 | 53.094 | 55.317 | 53.458 | 37.500 | 585 | No report | 13, 14 | 54.97 | 100.00 | No report | No report | 19.66 | High | Anxiety symptoms | Secondary | Physical activity | Change in frequency | No | Non-validated survey |
|  |  |  |  |  |  |  |  |  |  |  |  |  |  |  |  |  |  |  |  |  |  |  | Primary | Sleep | Change in frequency | No | Non-validated survey |
|  |  |  |  |  |  |  |  |  |  |  |  |  |  |  |  |  |  |  |  |  |  |  | Secondary | Leisure activities | Change in frequency | Yes | Non-validated survey |
| Peñaranda et al. (2022) | 15 | Observational | Cross-sectional | N/A | Colombia | 18 | 15887.200 | 338.878 | 64.205 | 62.655 | 60.247 | 75.000 | 1,345 | 47.12 | No report | 66.17 | No report | 37.10 | 0.00 | 62.60 | Middle | Depressive symptoms | Secondary | Work/studies | Change in frequency | No | Non-validated survey |
|  |  |  |  |  |  |  |  |  |  |  |  |  |  |  |  |  |  |  |  |  |  | Anxiety symptoms | Secondary | Work/studies | Change in frequency | No | Non-validated survey |
|  |  |  |  |  |  |  |  |  |  |  |  |  |  |  |  |  |  |  |  |  |  | General psychological distress | Secondary | Work/studies | Change in frequency | No | Non-validated survey |
| Pensgaard et al. (2021) | 15 | Observational | Cross-sectional | N/A | Norway | 9 | 597.500 | 1.840 | 36.980 | 36.907 | 36.110 | 37.500 | 378 | 26.86 | 18, 59 | 42.06 | No report | No report | No report | No report | High | Depressive symptoms | N/A | Unspecified generic routines | Regularity | No | "Yes/No" |
|  |  |  |  |  |  |  |  |  |  |  |  |  |  |  |  |  |  |  |  |  |  | Anxiety symptoms | N/A | Unspecified generic routines | Regularity | No | "Yes/No" |
| Rens et al. (2021) | 15 | Observational | Cross-sectional | N/A | Belgium | 4 | 3076.461 | 571.470 | 66.060 | 62.997 | 81.480 | 87.500 | 2,008 | 21.84 | 16, 25 | 78.09 | No report | No report | No report | No report | High | General psychological distress | Secondary | Leisure activities | Change in frequency | Yes | Non-validated survey |
|  |  |  |  |  |  |  |  |  |  |  |  |  |  |  |  |  |  |  |  |  |  |  |  |  |  | No |  |
|  |  |  |  |  |  |  |  |  |  |  |  |  |  |  |  |  |  |  |  |  |  |  | Secondary | Social activities | Change in frequency | No | Non-validated survey |
|  |  |  |  |  |  |  |  |  |  |  |  |  |  |  |  |  |  |  |  |  |  |  | Primary | Home activities | Change in frequency | No | "Yes/No" |
| Ryu et al. (2021) | 14 | Observational | Cross-sectional | N/A | Spain | 5 | 486.558 | 72.015 | 66.735 | 64.170 | 78.584 | 84.677 | 142 | 45.00 | No report | 69.72 | No report | 54.93 | No report | No report | High | Anxiety symptoms | Secondary | Social activities | Change in frequency | No | Non-validated survey |
| Sato et al. (2021) | 16 | Observational | Cross-sectional | N/A | Japan | 5 | 22.291 | 3.839 | 49.142 | 45.449 | 41.994 | 75.000 | 2,846 | 50.30 | No report | 59.59 | No report | No report | No report | No report | High | Depressive symptoms | Secondary | Physical activity | Change in frequency | No | "Yes/No" |
|  |  |  |  |  |  |  |  |  |  |  |  |  |  |  |  |  |  |  |  |  |  |  | Secondary | Work/studies | Change in frequency | No | "Yes/No" |
|  |  |  |  |  |  |  |  |  |  |  |  |  |  |  |  |  |  |  |  |  |  |  | Primary | Home activities | Change in frequency | No | "Yes/No" |
| Schneider et al. (2023) | 15 | Observational | Cross-sectional | N/A | Germany | 26 | 58642.574 | 79.597 | 62.376 | 57.000 | 38.665 | 100.000 | 2,828 | 47.30 | No report | 54.67 | No report | No report | No report | No report | High | Depressive symptoms | N/A | Unspecified generic routines | Change in capability | No | Non-validated survey |
|  |  |  |  |  |  |  |  |  |  |  |  |  |  |  |  |  |  |  |  |  |  | Anxiety symptoms | N/A | Unspecified generic routines | Change in capability | No | Non-validated survey |
| Şentürk et al. (2021) | 13 | Observational | Cross-sectional | N/A | Turkey | 12 | 9301.987 | 83.008 | 72.205 | 70.017 | 69.254 | 87.500 | 459 | 35.64 | 24, 60 | 44.66 | 0.00 | No report | 0.00 | No report | High | Depressive symptoms | Secondary | Work/studies | Change in frequency | No | Non-validated survey |
|  |  |  |  |  |  |  |  |  |  |  |  |  |  |  |  |  |  |  |  |  |  |  | Primary | Home activities | Change in frequency | No | Non-validated survey |
|  |  |  |  |  |  |  |  |  |  |  |  |  |  |  |  |  |  |  |  |  |  | Anxiety symptoms | Secondary | Work/studies | Change in frequency | No | Non-validated survey |
|  |  |  |  |  |  |  |  |  |  |  |  |  |  |  |  |  |  |  |  |  |  |  | Primary | Home activities | Change in frequency | No | Non-validated survey |
|  |  |  |  |  |  |  |  |  |  |  |  |  |  |  |  |  |  |  |  |  |  | General psychological distress | Secondary | Work/studies | Change in frequency | No | Non-validated survey |
|  |  |  |  |  |  |  |  |  |  |  |  |  |  |  |  |  |  |  |  |  |  |  | Primary | Home activities | Change in frequency | No | Non-validated survey |
| Shatla et al. (2020) | 10 | Observational | Cross-sectional | N/A | Saudi Arabia | 5 | 1702.392 | 8.872 | 76.418 | 78.407 | 90.396 | 62.500 | 1,921 | No report | No report | 49.66 | 12.70 | No report | 20.61 | No report | High | Depressive symptoms | N/A | Unspecified generic routines | Regularity | No | "Yes/No" |
|  |  |  |  |  |  |  |  |  |  |  |  |  |  |  |  |  |  |  |  |  |  | Anxiety symptoms | N/A | Unspecified generic routines | Regularity | No | "Yes/No" |
| Shoshani et al. (2022) | 15 | Observational | Cross-sectional | N/A | Israel | 5 | 131.545 | 7.299 | 73.437 | 69.643 | 77.301 | 100.000 | 1,537 | 13.97 | 11, 17 | 51.98 | 100.00 | No report | No report | No report | High | General psychological distress | N/A | Combined multiple routines | Regularity | No | Validated instrument |
| Sommerlad et al. (2022) | 14 | Observational | Cross-sectional | N/A | United Kingdom | 8 | 491.473 | 8.372 | 69.069 | 64.652 | 67.636 | 100.000 | 71,117 | 49.10 | No report | 75.03 | 32.99 | 69.98 | 34.85 | No report | High | Depressive symptoms | Secondary | Social activities | Change in frequency | Yes | Non-validated survey |
|  |  |  |  |  |  |  |  |  |  |  |  |  |  |  |  |  |  |  |  |  |  |  |  |  |  | No |  |
| Stanton et al. (2020) | 13 | Observational | Cross-sectional | N/A | Australia | 4 | 91.184 | 2.824 | 61.425 | 59.485 | 71.110 | 75.000 | 1,491 | 50.50 | No report | 67.00 | No report | 37.17 | No report | 46.48 | High | Depressive symptoms | Secondary | Physical activity | Change in frequency | No | Validated instrument |
|  |  |  |  |  |  |  |  |  |  |  |  |  |  |  |  |  |  |  |  |  |  |  | Primary | Sleep | Change in capability | No | Non-validated survey |
|  |  |  |  |  |  |  |  |  |  |  |  |  |  |  |  |  |  |  |  |  |  |  | Secondary | Smoking | Change in frequency | No | Non-validated survey |
|  |  |  |  |  |  |  |  |  |  |  |  |  |  |  |  |  |  |  |  |  |  |  | Secondary | Alcohol | Change in frequency | No | Validated instrument |
|  |  |  |  |  |  |  |  |  |  |  |  |  |  |  |  |  |  |  |  |  |  | Anxiety symptoms | Secondary | Physical activity | Change in frequency | No | Validated instrument |
|  |  |  |  |  |  |  |  |  |  |  |  |  |  |  |  |  |  |  |  |  |  |  | Primary | Sleep | Change in capability | No | Non-validated survey |
|  |  |  |  |  |  |  |  |  |  |  |  |  |  |  |  |  |  |  |  |  |  |  | Secondary | Smoking | Change in frequency | No | Non-validated survey |
|  |  |  |  |  |  |  |  |  |  |  |  |  |  |  |  |  |  |  |  |  |  |  | Secondary | Alcohol | Change in frequency | No | Validated instrument |
|  |  |  |  |  |  |  |  |  |  |  |  |  |  |  |  |  |  |  |  |  |  | General psychological distress | Secondary | Physical activity | Change in frequency | No | Validated instrument |
|  |  |  |  |  |  |  |  |  |  |  |  |  |  |  |  |  |  |  |  |  |  |  | Primary | Sleep | Change in capability | No | Non-validated survey |
|  |  |  |  |  |  |  |  |  |  |  |  |  |  |  |  |  |  |  |  |  |  |  | Secondary | Smoking | Change in frequency | No | Non-validated survey |
|  |  |  |  |  |  |  |  |  |  |  |  |  |  |  |  |  |  |  |  |  |  |  | Secondary | Alcohol | Change in frequency | No | Validated instrument |
| Sum et al. (2023) | 13 | Observational | Cross-sectional | N/A | Hong Kong SAR | 8 | 205.186 | 4.278 | 73.035 | 69.179 | 66.670 | 100.000 | 1,020 | 21.60 | 16, 30 | 72.94 | 0.00 | 98.32 | No report | No report | High | Depressive symptoms | Primary | Sleep | Change in frequency | No | Non-validated survey |
|  |  |  |  |  |  |  |  |  |  |  |  |  |  |  |  |  |  |  |  |  |  |  | Secondary | Work/studies | Change in frequency | No | Non-validated survey |
| Tanaka et al. (2022) | 18 | Observational | Cross-sectional | N/A | Japan | 15 | 338.842 | 10.109 | 56.458 | 50.236 | 44.115 | 100.000 | 83 | 58.60 | No report | 55.42 | No report | No report | 43.37 | 46.99 | High | Depressive symptoms | Secondary | Physical activity | Change in frequency | No | Validated instrument |
| Tanikaga et al. (2023) | 16 | Observational | Cross-sectional | N/A | Japan | 10 | 140.234 | 1.538 | 50.520 | 43.450 | 35.190 | 100.000 | 74 | 78.60 | No report | 45.95 | No report | No report | No report | No report | High | Depressive symptoms | Secondary | Leisure activities | Change in frequency | No | Validated instrument |
|  |  |  |  |  |  |  |  |  |  |  |  |  |  |  |  |  |  |  |  |  |  |  | Secondary | Social activities | Change in frequency | No | Validated instrument |
|  |  |  |  |  |  |  |  |  |  |  |  |  |  |  |  |  |  |  |  |  |  |  | N/A | Combined multiple routines | Change in frequency | No | Validated instrument |
| Tondokoro et al. (2023) | 17 | Observational | Cross-sectional | N/A | Japan | 12 | 674.004 | 10.448 | 54.891 | 48.441 | 42.949 | 100.000 | 25,762 | 47.01 | No report | 49.03 | 27.00 | 43.91 | 0.00 | No report | High | General psychological distress | Primary | Sleep | Change in frequency | No | Non-validated survey |
|  |  |  |  |  |  |  |  |  |  |  |  |  |  |  |  |  |  |  |  |  |  |  | Secondary | Work/studies | Change in frequency | No | Non-validated survey |
| Valdés et al. (2022) | 14 | Observational | Cross-sectional | N/A | Chile | 9 | 2618.177 | 75.547 | 80.782 | 78.870 | 87.500 | 94.167 | 5,037 | 21.45 | No report | 70.40 | No report | No report | 88.03 | No report | High | Depressive symptoms | N/A | Unspecified generic routines | Regularity | No | Non-validated survey |
|  |  |  |  |  |  |  |  |  |  |  |  |  |  |  |  |  |  |  |  |  |  | Anxiety symptoms | N/A | Unspecified generic routines | Regularity | No | Non-validated survey |
|  |  |  |  |  |  |  |  |  |  |  |  |  |  |  |  |  |  |  |  |  |  | General psychological distress | N/A | Unspecified generic routines | Regularity | No | Non-validated survey |
| Vrublevska et al. (2022) | 14 | Observational | Cross-sectional | N/A | Italy | 7 | 113.861 | 6.573 | 67.113 | 65.986 | 66.993 | 75.000 | 2,608 | No report | No report | 60.28 | 39.92 | 33.55 | 54.71 | No report | High | Anxiety symptoms | Secondary | Leisure activities | Change in frequency | Yes | "Yes/No" |
|  |  |  |  |  |  |  |  |  |  |  |  |  |  |  |  |  |  |  |  |  |  |  | Secondary | Leisure activities | Change in frequency | Yes | Non-validated survey |
|  |  |  |  |  |  |  |  |  |  |  |  |  |  |  |  |  |  |  |  |  |  |  | N/A | Unspecified generic routines | Regularity | No | "Yes/No" |
| Wang et al. (2022) | 14 | Observational | Cross-sectional | N/A | China | 22 | 0.984 | 0.005 | 79.026 | 81.388 | 78.453 | 62.500 | 8112 | No report | 20, 60 | 86.50 | 57.78 | 4.98 | 40.80 | No report | Middle | Depressive symptoms | Secondary | Physical activity | Change in capability | No | Non-validated survey |
|  |  |  |  |  |  |  |  |  |  |  |  |  |  |  |  |  |  |  |  |  |  |  | Primary | Eating | Change in capability | No | Non-validated survey |
|  |  |  |  |  |  |  |  |  |  |  |  |  |  |  |  |  |  |  |  |  |  | Anxiety symptoms | Secondary | Physical activity | Change in capability | No | Non-validated survey |
|  |  |  |  |  |  |  |  |  |  |  |  |  |  |  |  |  |  |  |  |  |  |  | Primary | Eating | Change in capability | No | Non-validated survey |
| Watson et al. (2023) | 16 | Observational | Cross-sectional | N/A | US | 16 | 5416.071 | 61.390 | 61.204 | 61.018 | 54.531 | 62.500 | 56 | 13.29 | 10, 17 | 55.36 | No report | No report | No report | No report | High | Depressive symptoms | N/A | Unspecified generic routines | Regularity | No | Validated instrument |
|  |  |  |  |  |  |  |  |  |  |  |  |  |  |  |  |  |  |  |  |  |  | Anxiety symptoms | N/A | Unspecified generic routines | Regularity | No | Validated instrument |
| Wu et al. (2023) | 9 | Observational | Cross-sectional | N/A | China | 29 | 1352.326 | 1.141 | 81.770 | 84.520 | 79.170 | 62.500 | 6,154 | No report | 33, 49 | 63.41 | No report | No report | No report | No report | Middle | Depressive symptoms | Secondary | Physical activity | Change in capability | No | Validated instrument |
|  |  |  |  |  |  |  |  |  |  |  |  |  |  |  |  |  |  |  |  |  |  |  | Primary | Eating | Change in capability | No | Validated instrument |
|  |  |  |  |  |  |  |  |  |  |  |  |  |  |  |  |  |  |  |  |  |  |  | Primary | Sleep | Change in capability | No | Validated instrument |
|  |  |  |  |  |  |  |  |  |  |  |  |  |  |  |  |  |  |  |  |  |  |  | Primary | Home activities | Change in capability | No | Validated instrument |
| Yılmaz & Önal. (2023) | 16 | Observational | Cross-sectional | N/A | Turkey | 17 | 5322.292 | 89.852 | 74.938 | 78.500 | 74.955 | 50.000 | 1,320 | 71.03 | 65, 99 | 53.18 | 95.15 | 22.80 | 93.26 | 70.45 | High | Depressive symptoms | Primary | Eating | Regularity | No | Non-validated survey |
|  |  |  |  |  |  |  |  |  |  |  |  |  |  |  |  |  |  |  |  |  |  |  | Primary | Sleep | Regularity | No | Non-validated survey |

Note.

**Study quality.** Assessed by the Appraisal Tool for Cross-Sectional Studies (AXIS; Downes et al., 2016).

**COVID-19 severity (incidence and death).** The unit is monthly cumulative per million individuals. Information was extracted from official websites.

**Physical comorbidity.** The proportion (%) reflects at least what proportion of the sample had a known physical disease (based on ICD-11 categories).

**Country-level income.** Categories were assigned based on the cut-offs for GNI per capita suggested by The World Bank: Low = $1,085 or less; Middle = $1,086 to $13,204; High = $13,205 or more.

**Routines.** ***Primary routines*** included eating, sleep, and home activities; ***Secondary routines*** included physical activity, leisure activities, social activities, and work/studies (Hou et al., 2018; Hou et al., 2019). Validated scales (including any modified version) included the following: ***physical activity*** – COVID-19 Exposure and Family Impact Scales (CEFIS), International Physical Activity Questionnaire (IPAQ), Physical Activity and Sedentary Behaviours–Short Form (IPAD-SF), Physical Activity Vital Sign; ***eating*** – COVID-19 Exposure and Family Impact Scales (CEFIS); ***sleep*** – COVID-19 Exposure and Family Impact Scales (CEFIS), Pittsburg Sleep Quality Index (PSQI); ***leisure activities*** – Activity Card Sort, Coronavirus Perinatal Experiences–Impact Survey; ***social activities*** – Activity Card Sort, Index for the Assessment of Health Impairments, Coronavirus Perinatal Experiences–Impact Survey; ***work/studies*** – Child Routines Inventory (CRI), Pandemic WorkConflict Scale (PWCS); ***home activities*** – COVID-19 Exposure and Family Impact Scales (CEFIS), Child Routines Inventory (CRI); ***combined multiple routines*** – Activity Card Sort, Adolescent Routines Questionnaire (ARQ), Brief Social Rhythm Scale – 10 (BSRS), Sustainability of Living Inventory – 2 (SOLI-2); ***unspecified generic routines*** – Child Routines Inventory (CRI), Pandemic Stress Questionnaire; ***alcohol*** – Alcohol Use Disorders Identification Test (AUDIT).

**Psychiatric symptoms.** Validated scales included: ***depressive symptoms*** *–* Center for Epidemiologic Studies Depression Scale (CES-D), Center for Epidemiologic Studies Depression Scale for Children (CES-DC), Depression Anxiety Stress Scale–21 (DASS-21) Depression Subscale, Edinburgh Postnatal Depression Scale (EPDS), Geriatric Depression Scale–15 (GDS-15), Geriatric Depression Scale–30 (GDS-30), Hopkins Symptoms Checklist–10 (HSCL-10) Depression Subscale, Hospital Anxiety and Depression Scale (HADS) Depression Subscale, Patient Health Questionnaire–2 (PHQ-2), Patient Health Questionnaire–9 (PHQ-9), Patient-Reported Outcomes Measurement Information System (PROMIS) Depression Subscale, Zung Self-Rating Depression Scale (SDS); ***anxiety symptoms*** *–* Depression Anxiety Stress Scale–21 (DASS-21) Anxiety Subscale, Generalized Anxiety Disorder–2 (GAD-2), Generalized Anxiety Disorder–7 (GAD-7), Hopkins Symptoms Checklist–10 (HSCL-10) Anxiety Subscale, Hospital Anxiety and Depression Scale (HADS) Anxiety Subscale, Patient-Reported Outcomes Measurement Information System (PROMIS) Anxiety Subscale, Screen for Child Anxiety Related Disorders (SCARED), Spence Children’s Anxiety Scale–Short Form (SCAS-SF), State-Trait Anxiety Inventory (STAI), Zung Self-Rating Anxiety Scale (SAS); ***general psychological distress*** *–* Brief Symptom Inventory–18 (BSI-18), Depression Anxiety Stress Scale–21 (DASS-21) Stress Subscale, General Health Questionnaire–12 (GHQ-12), General Health Questionnaire–28 (GHQ-28), Kessler Psychological Distress Scale–6 (K6), Perceived Stress Scale–10 (PSS-10); ***posttraumatic stress disorder (PTSD) symptoms*** *–* Impact of Events Scale–Revised (IES-R), Primary Care PTSD Screen for DSM-5 (PC-PTSD-5), PTSD Checklist for DSM-5 (PCL-5); and ***depressive and anxiety symptoms combined*** – Aga Khan University Anxiety and Depression Scale (AKUADS), Hospital Anxiety and Depression Scale (HADS), Patient Health Questionnaire–4 (PHQ-4) (i.e., Patient Health Questionnaire–2 + Generalized Anxiety Disorder–2).

**REFERENCES (FOOTNOTES)**

Downes, M. J., Brennan, M. L., Williams, H. C., & Dean, R. S. (2016). Development of a critical appraisal tool to assess the quality of cross-sectional studies (AXIS). *BMJ Open, 6*(12), e011458.

Hou, W. K., Hall, B. J., & Hobfoll, S. E. (2018). Drive to thrive: A theory of resilience following loss. In N. Morina & A. Nickerson (Eds.), *Mental health of refugee and conflict-affected populations: Theory, research and clinical practice* (pp. 111-133). Springer.

Hou, W. K., Lai, F. T. T., Hougen, C., Hall, B. J., & Hobfoll, S. E. (2019). Measuring everyday processes and mechanisms of stress resilience: Development and initial validation of the Sustainability of Living Inventory (SOLI). *Psychological Assessment, 31*(6), 715-729.

The World Bank. (n.d.) World Bank country and lending groups. *The World Bank*. Retrieved from https://datahelpdesk.worldbank.org/knowledgebase/articles/906519-world-bank-country-and-lending-groups#:~:text=%EF%BB%BF%EF%BB%BF%20For%20the%20current,those%20with%20a%20GNI%20per

**REFERNECES (53 INCLUDED STUDIES)**

Aolymat, I., Khasawneh, A. I., & Al-Tamimi, M. (2022). COVID-19-associated mental health impact on menstrual function aspects: Dysmenorrhea and premenstrual syndrome, and genitourinary tract health: A cross sectional study among Jordanian medical students. *International Journal of Environmental Research and Public Health, 19*(3), 1439. https://doi.org/10.3390/ijerph19031439

Candela, C. F., Pia, L. J., Pons-Fuster, E., & Tvarijonaviciute, A. (2022). Impact of the COVID-19 pandemic upon patients with burning mouth syndrome. *Journal of Stomatology, Oral and Maxillofacial Surgery, 123*(2), 101-104. https://doi.org/10.1016/j.jormas.2021.07.001

Cho, S., Ju, H. R., Oh, H., Choi, E. S., & Lee, J. A. (2022). The association between the restriction of daily life and depression during the COVID-19 pandemic in Korea: A nationwide based survey. *Scientific Reports, 12*(1), 17722. https://doi.org/10.1038/s41598-022-21301-5

Czenczek-Lewandowska, E., Wyszyńska, J., Leszczak, J., Baran, J., Weres, A., Mazur, A., & Lewandowski, B. (2021). Health behaviours of young adults during the outbreak of the COVID-19 pandemic–a longitudinal study. *BMC Public Health, 21*, 1038. https://doi.org/10.1186/s12889-021-11140-w

Fila-Witecka, K., Senczyszyn, A., Kołodziejczyk, A., Ciułkowicz, M., Maciaszek, J., Misiak, B., Szcześniak, D., & Rymaszewska, J. (2021). Lifestyle changes among Polish nniversity students during the COVID-19 pandemic. *International Journal of Environmental Research and Public Health, 18*(18), 9571. https://doi.org/10.3390/ijerph18189571

Gómez-Baya, D., Gómez-Gómez, I., Domínguez-Salas, S., Rodríguez-Domínguez, C., & Motrico, E. (2022). The influence of lifestyles to cope with stress over mental health in pregnant and postpartum women during the COVID-19 pandemic. *Current Psychology*. https://doi.org/10.1007/s12144-022-03287-5

Hampshire, A., Hellyer, P. J., Soreq, E., Mehta, M. A., Ioannidis, K., Trender, W., Grant, J. E., & Chamberlain, S. R. (2021). Associations between dimensions of behaviour, personality traits, and mental-health during the COVID-19 pandemic in the United Kingdom. *Nature Communications, 12*(1), 4111. https://doi.org/10.1038/s41467-021-24365-5

Heesen, G., Heinemann, S., Müller, F., Dopfer-Jablonka, A., Mikuteit, M., Niewolik, J., Klawonn, F., Vlahdiek, K., Hummers, E., & Schröder, D. (2022). Social participation and mental health of immunocompromised individuals before and after COVID-19 vaccination–Results of a longitudinal observational study over three time points. *Frontiers in Psychiatry, 13*, 1080106. https://doi.org/10.3389/fpsyt.2022.1080106

Ho, H. C., Chui, O. S., & Chan, Y. C. (2022). When pandemic interferes with work: Psychological capital and mental health of social workers during COVID-19. *Social Work, 67*(4), 311-320. https://doi.org/10.1093/sw/swac035

Hou, W. K., Tong, H., Liang, L., Li, T. W., Liu, H., Ben-Ezra, M., Goodwin, R., & Lee, T. M. C. (2021). Probable anxiety and components of psychological resilience amid COVID-19: A population-based study. *Journal of Affective Disorders, 282*, 594-601. https://doi.org/10.1016/j.jad.2020.12.127

Inam, Q.-U.-A., Shaikh, S., Wahab, S., Ovais, M. H., Memon, U. A. A., & Anwar, Z. (2022). Psychological impact of COVID-19 Pandemic and associated factors on college students. *The Journal of the Pakistan Medical Association, 72*(10), 2014-2018. https://doi.org/10.47391/JPMA.4321

Jang, H. Y., Ko, Y., & Han, S. Y. (2022). Factors associated with depressive symptoms in individuals who have experienced COVID-19 self-quarantine. *Frontiers in Public Health, 10*, 810475. https://doi.org/10.3389/fpubh.2022.810475

Kahawage, P., Bullock, B., Meyer, D., Gottlieb, J., Crowe, M., Swartz, H. A., Yatham, L. N., Inder, M., Porter, R. J., Nierenberg, A. A., Meesters, Y., Gordjin,M., Haarman, B. C. M., & Murray, G. (2022). Social rhythm disruption is associated with greater depressive symptoms in people with mood disorders: Findings from a multinational online survey during COVID-19. *The Canadian Journal of Psychiatry, 67*(11), 832-841. https://doi.org/10.1177/07067437221097905

Kim, S. W., Park, I. H., Kim, M., Park, A. L., Jhon, M., Kim, J. W., Kang, H. J., Ryu, S., Lee, J. Y., & Kim, J. M. (2021). Risk and protective factors of depression in the general population during the COVID-19 epidemic in Korea. *BMC Psychiatry, 21*, 445. https://doi.org/10.1186/s12888-021-03449-y

Kornilaki, E. N. (2022). The psychological effect of COVID‐19 quarantine on Greek young adults: Risk factors and the protective role of daily routine and altruism. *International Journal of Psychology, 57*(1), 33-42. https://doi.org/10.1002/ijop.12767

Kua, Z., Hamzah, F., Tan, P. T., Ong, L. J., Tan, B., & Huang, Z. (2022). Physical activity levels and mental health burden of healthcare workers during COVID‐19 lockdown. *Stress and Health, 38*(1), 171-179. https://doi.org/10.1002/smi.3078

Lai, F. T. T., Chan, V. K. Y., Li, T. W., Li, X., Hobfoll, S. E., Lee, T. M. C., & Hou, W. K. (2022). Disrupted daily routines mediate the socioeconomic gradient of depression amid public health crises: A repeated cross-sectional study. *Australian & New Zealand Journal of Psychiatry, 56*(10), 1320-1331. https://doi.org/10.1177/00048674211051271

Lee, H., & Chu, H. S. (2022). The effects of changes in daily life due to the COVID‐19 pandemic on the depressive symptoms among community‐dwelling older adults in Korea. *International Journal of Mental Health Nursing, 31*(4), 974-984. https://doi.org/10.1111/inm.13008

Lee, S. B., Jeon, Y. B., & Yoon, M. S. (2022). Dual mediating effects of changes in daily life and anxiety on the relationship between occupation and depression in Korea during the COVID-19 pandemic. *BMC Public Health, 22*, 1492. https://doi.org/10.1186/s12889-022-13932-0

Li, G., Liu, H., Qiu, C., & Tang, W. (2022). Fear of COVID-19, prolonged smartphone use, sleep disturbances, and depression in the time of COVID-19: A nation-wide survey. *Frontiers in Psychiatry, 13*, 971800. https://doi.org/10.3389/fpsyt.2022.971800

Li, T. W., Liang, L., Ho, P. L., Yeung, E. T. F., Hobfoll, S. E., & Hou, W. K. (2022). Coping resources mediate the prospective associations between disrupted daily routines and persistent psychiatric symptoms: A population-based cohort study. *Journal of Psychiatric Research, 152*, 260-268. https://doi.org/10.1016/j.jpsychires.2022.05.033

Lin, P., Hillstrom, K., Gottesman, K., Jia, Y., Kuo, T., & Robles, B. (2023). Financial and other life stressors, psychological distress, and food and beverage consumption among students attending a large California state university during the COVID-19 pandemic. *International Journal of Environmental Research and Public Health, 20*(4), 3668. https://doi.org/10.3390/ijerph20043668

Liu, J., Zhou, T., Yuan, M., Ren, H., Bian, X., & Coplan, R. J. (2021). Daily routines, parent–child conflict, and psychological maladjustment among Chinese children and adolescents during the COVID-19 pandemic. *Journal of Family Psychology, 35*(8), 1077-1085. https://doi.org/10.1037/fam0000914

Lotzin, A., Krause, L., Acquarini, E., Ajdukovic, D., Anastassiou-Hadjicharalambous, X., Ardino, V., Bondjers, K., Böttche, M., Dragan, M., Figueiredo-Braga, M., Gelezelyte, O., Grajewski, P., Javakhishvili, J. D., Kazlauskas, E., Lenferink, L., Lioupi, C., Lueger-Schuster, B., Mooren, T., Sales, L., … & ADJUST Study Consortium. (2022). Risk and protective factors for posttraumatic stress disorder in trauma-exposed individuals during the COVID-19 pandemic–findings from a pan-European study. *European Journal of Psychotraumatology, 13*(2), 2138099. https://doi.org/10.1080/20008066.2022.2138099

Martinelli, N., Gil, S., Belletier, C., Chevalère, J., Dezecache, G., Huguet, P., & Droit-Volet, S. (2021). Time and emotion during lockdown and the Covid-19 epidemic: Determinants of our experience of time?. *Frontiers in Psychology, 11*, 616169. https://doi.org/10.3389/fpsyg.2020.616169

Matsuo, M., Sesoko, S., Kosa, A., Noda, S., Koura, S., Miyabara, H., & Higuchi, T. (2022). Factors affecting the mental health of medical students during the COVID-19 pandemic: A cross-sectional study. *Medicine, 101*(47), e31897. https://doi.org/10.1097/MD.0000000000031897

McGoron, L., Wargo Aikins, J., Trentacosta, C. J., Gómez, J. M., & Beeghly, M. (2022). School support, chaos, routines, and parents’ mental health during COVID-19 remote schooling. *School Psychology, 37*(2), 173-182. https://doi.org/10.1037/spq0000467

McMahon, G., Douglas, A., Casey, K., & Ahern, E. (2022). Disruption to well-being activities and depressive symptoms during the COVID-19 pandemic: The mediational role of social connectedness and rumination. *Journal of Affective Disorders, 309*, 274-281. https://doi.org/10.1016/j.jad.2022.04.142

Nyberg, G., Helgadóttir, B., Kjellenberg, K., & Ekblom, Ö. (2023). COVID-19 and unfavorable changes in mental health unrelated to changes in physical activity, sedentary time, and health behaviors among Swedish adolescents: A longitudinal study. *Frontiers in Public Health, 11*, 1115789. https://doi.org/10.3389/fpubh.2023.1115789

Peñaranda, A., García, E., Pérez-Herrera, L. C., Trojan, A., Peñaranda, D., Molina, J., & Moreno-López, S. (2022). Effect of the COVID-19 pandemic on the mental health, daily and occupational activities among health professionals in Colombia: A national study. *BMC Psychiatry,* 22, 682. https://doi.org/10.1186/s12888-022-04337-9

Pensgaard, A. M., Oevreboe, T. H., & Ivarsson, A. (2021). Mental health among elite athletes in Norway during a selected period of the COVID-19 pandemic. *BMJ Open Sport & Exercise Medicine, 7*, e001025. http://dx.doi.org/10.1136/bmjsem-2020-001025

Ren, H., He, X., Bian, X., Shang, X., & Liu, J. (2021). The protective roles of exercise and maintenance of daily living routines for Chinese adolescents during the COVID-19 quarantine period. *Journal of Adolescent Health, 68*(1), 35-42. https://doi.org/10.1016/j.jadohealth.2020.09.026

Rens, E., Smith, P., Nicaise, P., Lorant, V., & Van den Broeck, K. (2021). Mental distress and its contributing factors among young people during the first wave of COVID-19: A Belgian survey study. *Frontiers in Psychiatry, 12*, 575553. https://doi.org/10.3389/fpsyt.2021.575553

Ryu, J., Sükei, E., Norbury, A., H Liu, S., Campaña-Montes, J. J., Baca-Garcia, E., Artés, A., & Perez-Rodriguez, M. M. (2021). Shift in social media app usage during COVID-19 lockdown and clinical anxiety symptoms: Machine learning–based ecological momentary assessment study. *JMIR Mental Health, 8*(9), e30833. https://doi.org/10.2196/30833

Sato, K., Sakata, R., Murayama, C., Yamaguchi, M., Matsuoka, Y., & Kondo, N. (2021). Changes in work and life patterns associated with depressive symptoms during the COVID-19 pandemic: An observational study of health app (CALO mama) users. *Occupational and Environmental Medicine, 78*(9), 632-637. http://dx.doi.org/10.1136/oemed-2020-106945

Schneider, A., Huber, L., Lohse, J., Linde, K., Greissel, A., Sattel, H., Henningson, P., & Hapfelmeier, A. (2023). Association between somatic symptom disorder and symptoms with daily life impairment after SARS-CoV-2 infection-results from a population-based cross-sectional study. *Journal of Psychosomatic Research, 168*, 111230. https://doi.org/10.1016/j.jpsychores.2023.111230

Şentürk, E., Sağaltıcı, E., Geniş, B., & Günday Toker, Ö. (2021). Predictors of depression, anxiety and stress among remote workers during the COVID-19 pandemic. *Work, 70*(1), 41-51. https://doi.org/10.3233/WOR-210082

Shatla, M. M., Khafagy, A. A., Bulkhi, A. A., & Aljahdali, I. A. (2020). Public concerns and mental health changes related to the COVID-19 pandemic lockdown in Saudi Arabia. *Clin Lab, 66*(10), 2125-2132.

Shoshani, A., & Kor, A. (2022). The mental health effects of the COVID-19 pandemic on children and adolescents: Risk and protective factors. *Psychological Trauma: Theory, Research, Practice, and Policy, 14*(8), 1365-1373. https://doi.org/10.1037/tra0001188

Sommerlad, A., Marston, L., Huntley, J., Livingston, G., Lewis, G., Steptoe, A., & Fancourt, D. (2022). Social relationships and depression during the COVID-19 lockdown: Longitudinal analysis of the COVID-19 Social Study. *Psychological Medicine, 52*(15), 3381-3390. https://doi.org/10.1017/S0033291721000039

Stanton, R., To, Q. G., Khalesi, S., Williams, S. L., Alley, S. J., Thwaite, T. L., Fenning, A. S., & Vandelanotte, C. (2020). Depression, anxiety and stress during COVID-19: Associations with changes in physical activity, sleep, tobacco and alcohol use in Australian adults. *International Journal of Environmental Research and Public Health, 17*(11), 4065. https://doi.org/10.3390/ijerph17114065

Sum, M. Y., Wong, G. H. Y., & Chan, S. K. W. (2023). Depressive symptoms and its correlates in undergraduates during the COVID-19 pandemic. *East Asian Archives of Psychiatry, 33*(1), 21-27. https://search.informit.org/doi/10.3316/informit.010777136604681

Tanaka, S., Fujita, K., Yakushiji, K., Harada, N., & Yoshizumi, T. (2022). Changes in physical activity due to fear of COVID-19 and its impact on depression among post-liver transplant patients in Japan: A longitudinal survey study. *Annals of Transplantation, 27*, e938239-1. https://doi.org/10.12659/AOT.938239

Tanikaga, M., Uemura, J. I., Hori, F., Hamada, T., & Tanaka, M. (2023). Changes in community-dwelling elderly’s activity and participation affecting depression during COVID-19 pandemic: A cross-sectional study. *International Journal of Environmental Research and Public Health, 20*(5), 4228. https://doi.org/10.3390/ijerph20054228

Tao, T. J., Lee, T. M. C., Fung, A. L. C., Li, T. W., Ettman, C. K., Galea, S., & Hou, W. K. (2022). Low assets predict persistent depression through living difficulties amid large-scale disasters: A cohort study. *Journal of Affective Disorders, 315*, 282-290. https://doi.org/10.1016/j.jad.2022.07.040

Tondokoro, T., Nakata, A., Tateishi, S., Mafune, K., Tsuji, M., Ando, H., Odagami, K., Matsugaki, R., & Fujino, Y. (2023). Changes in work/sleep patterns due to the COVID-19 pandemic are associated with psychological distress among Japanese workers. *Frontiers in Psychology, 14*, 1133498. https://doi.org/10.3389/fpsyg.2023.1133498

Valdés, J. M., Díaz, F. J., Christiansen, P. M., Lorca, G. A., Solorza, F. J., Alvear, M., Ramírez, S., Nuñez, D., Araya, R., & Gaete, J. (2022). Mental health and related factors among undergraduate students during SARS-COV-2 pandemic: A cross-sectional study. *Frontiers in Psychiatry, 13*, 833263. https://doi.org/10.3389/fpsyt.2022.833263

Vrublevska, J., Perepjolkina, V., Martinsone, K., Kolesnikova, J., Krone, I., Smirnova, D., Fountoulakis, K. N., & Rancans, E. (2022). Determinants of anxiety in the general Latvian population during the COVID-19 state of emergency. *Frontiers in Public Health, 10*, 854812. https://doi.org/10.3389/fpubh.2022.854812

Wang, L., Zhang, H., Shang, C., Liang, H., Liu, W., Han, B., Xia, W., Zou, M., & Sun, C. (2022). Mental health issues in parents of children with autism spectrum disorder: A multi‐time‐point study related to COVID‐19 pandemic. *Autism Research, 15*(12), 2346-2358. https://doi.org/10.1002/aur.2836

Watson, K. H., Coiro, M. J., Ciriegio, A. E., Dakkak, A., Jones, M. T., Reisman, J., Kujawa, A., & Compas, B. E. (2023). COVID‐19 stressors and symptoms of anxiety and depression in a community sample of children and adolescents. *Child and Adolescent Mental Health, 28*(1), 172-179. https://doi.org/10.1111/camh.12598

Wu, J., Yang, H., Qin, Y., Wu, J., Yan, H., Xu, Y., Sun, X., Zhang, L., & Liu, X. (2023). Change of daily life and depression among adults under stringent lockdown restrictions during COVID-19 pandemic in Shanghai, China. *Asian Journal of Psychiatry, 79*, 103327. https://doi.org/10.1016/j.ajp.2022.103327

Yılmaz, B. A., & Önal, Ö. (2023). Effect of loneliness and sociodemographic, health, COVID-19 pandemic-related factors on depression among older adults. *Educational Gerontology, 49*(1), 12-26. https://doi.org/10.1080/03601277.2022.2065440

Yuan, M., Bian, X., Liu, J., Zhen, H., Coplan, R. J., & Sang, B. (2023). Relations between maternal panic over COVID-19 and children’s depressive symptoms: the moderating role of children’s daily routines. *Current Psychology*. https://doi.org/10.1007/s12144-022-04129-0
