# Supplementary material for: Daily routine disruptions and psychiatric symptoms amid COVID-19: a systematic review and meta-analysis of data from 0.9 million individuals in 32 countries
Source: BMC Med. 2024 Feb 2;22:49. doi: 10.1186/s12916-024-03253-x (PMC10835995; doi:10.1186/s12916-024-03253-x)
Supplement: Supplementary file 6 — Additional file 6: Supplementary Material 6. Forest plot for effect sizes of the association between daily routine disruptions (combined across types) and psychiatric symptoms. [file 12916_2024_3253_MOESM6_ESM.docx]

**SUPPLEMENTARY MATERIAL 6** Forest plot for effect sizes of the association between daily routine disruptions (combined across types) and psychiatric symptoms.

**Fig. 1** Forest plot for effect sizes of routine disruptions on depressive symptoms.

**
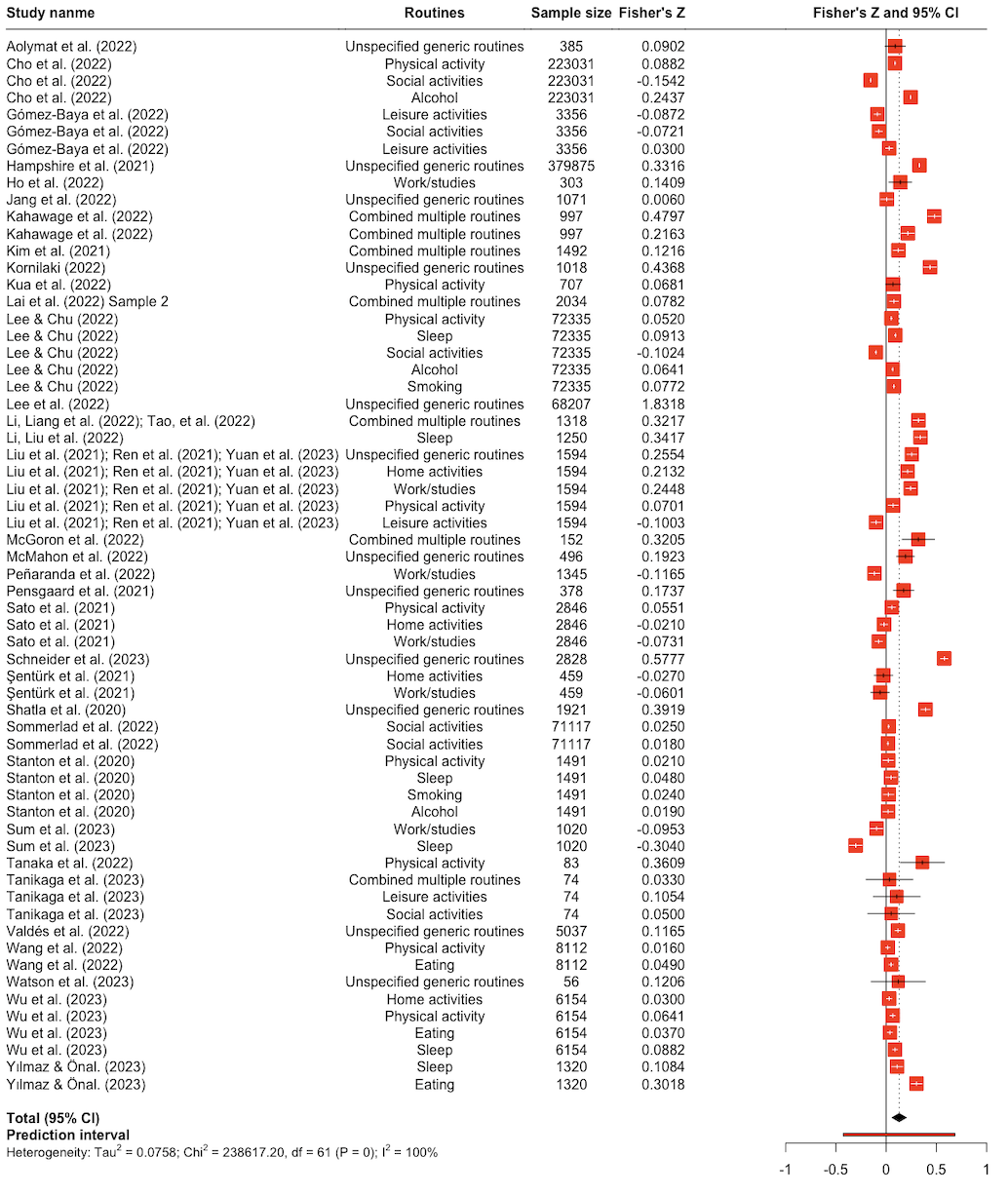
**

**Fig. 2** Forest plot for effect sizes of routine disruptions on anxiety symptoms.


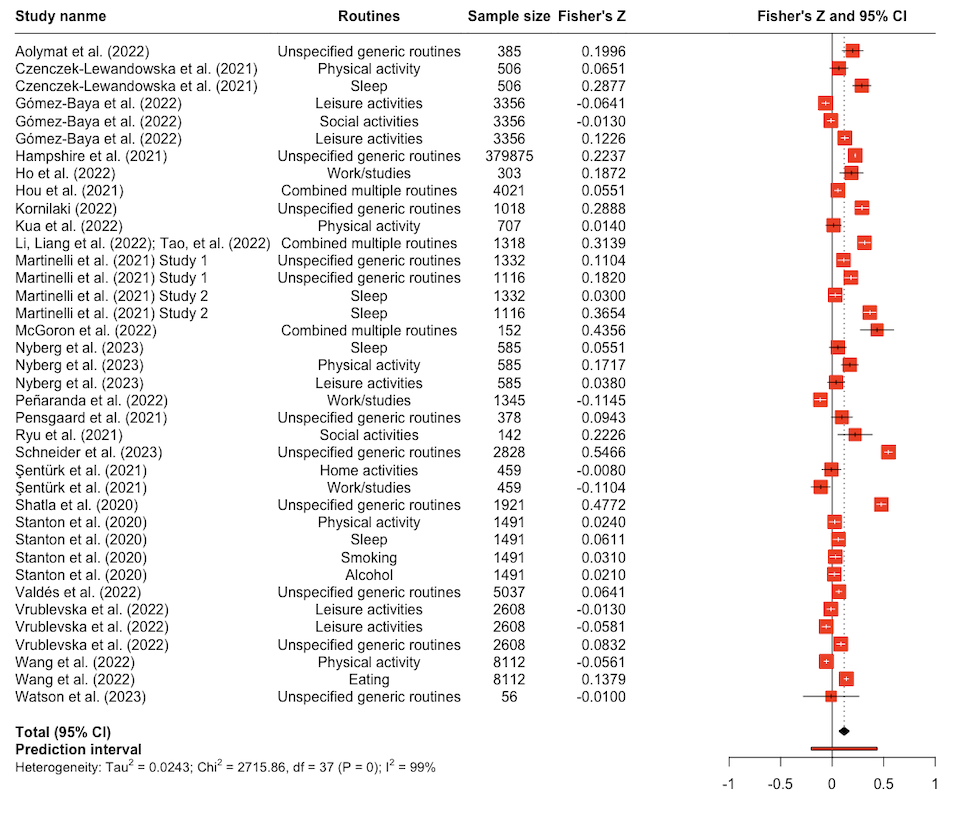


**Fig. 3** Forest plot for effect sizes of routine disruptions on posttraumatic stress disorder (PTSD) symptoms.

**
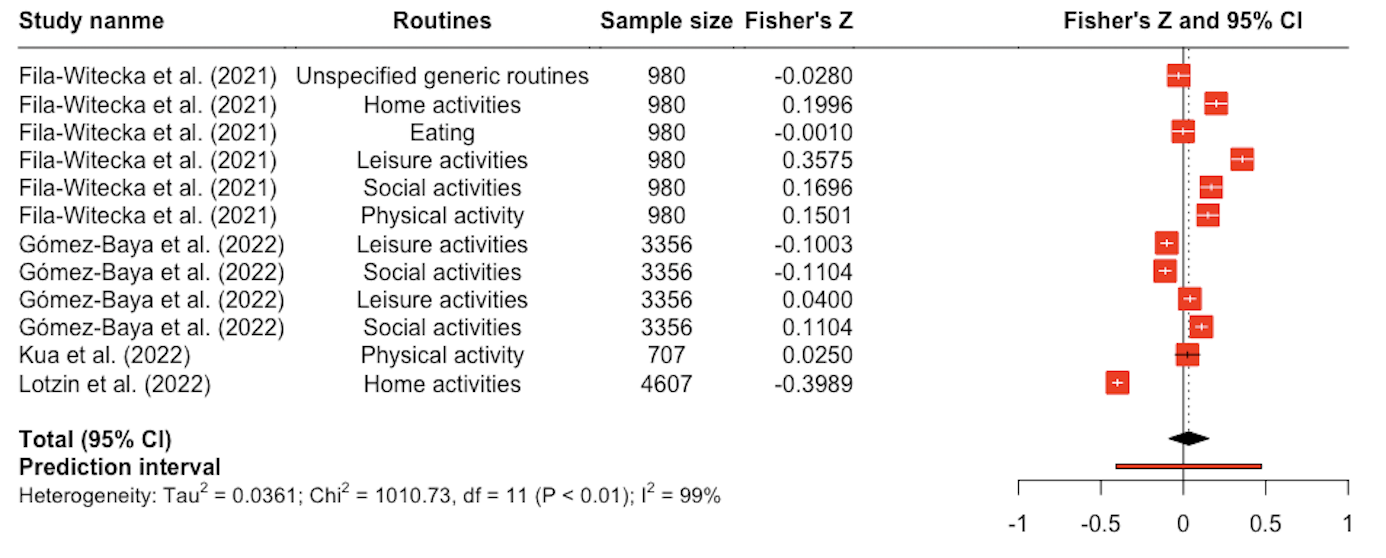
**

**Fig. 4** Forest plot for effect sizes of routine disruptions on depressive and anxiety symptoms.


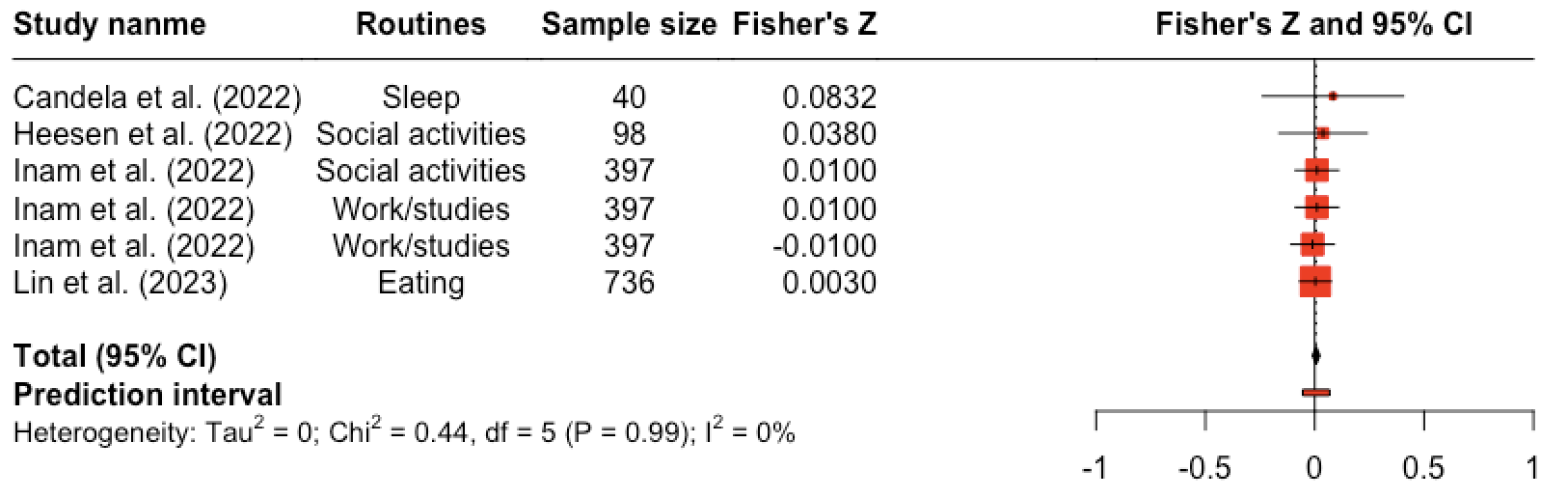


**Fig. 5** Forest plot for effect sizes of routine disruptions on general psychological distress.

**
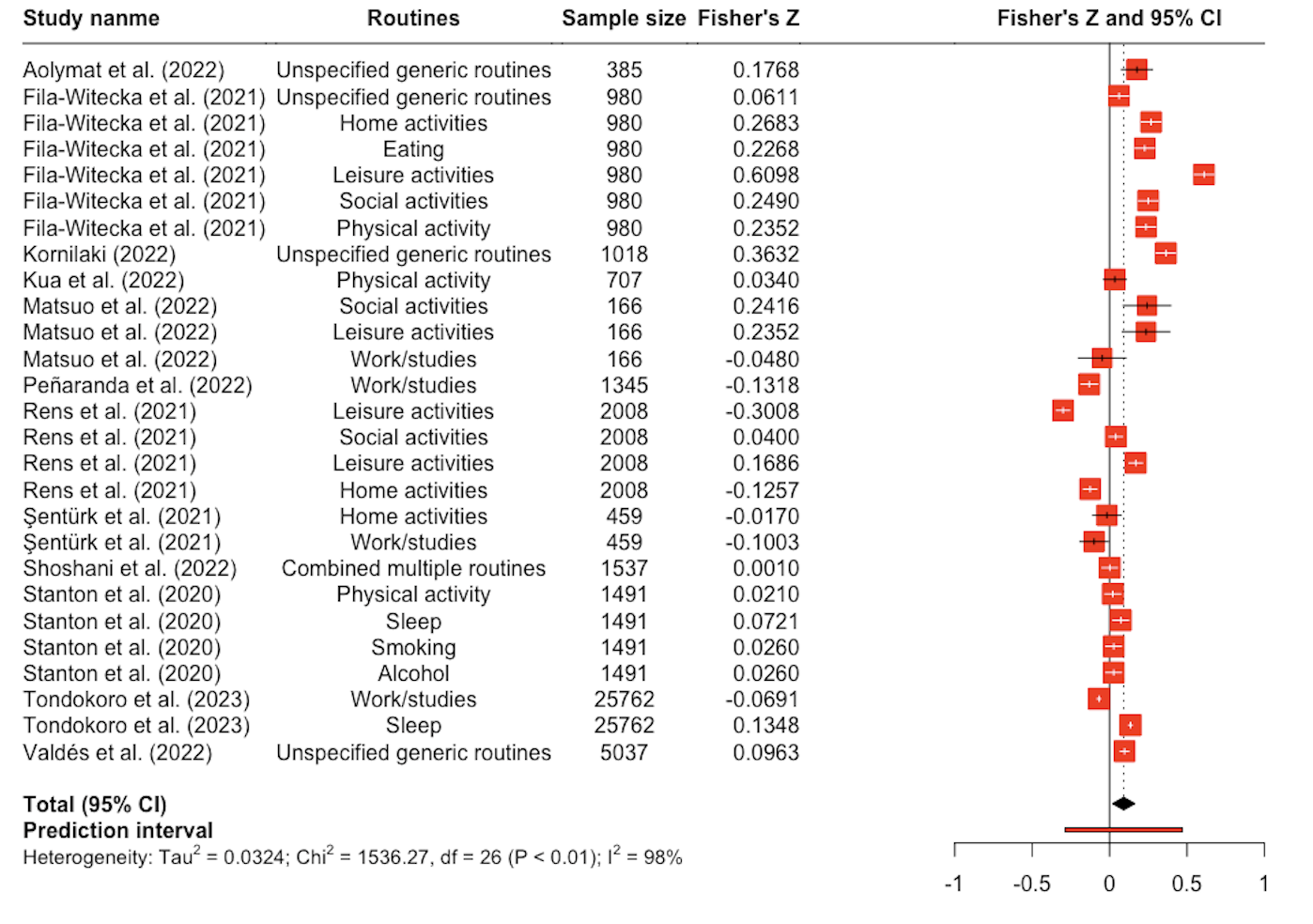
**
