# Supplementary material for: Daily routine disruptions and psychiatric symptoms amid COVID-19: a systematic review and meta-analysis of data from 0.9 million individuals in 32 countries
Source: BMC Med. 2024 Feb 2;22:49. doi: 10.1186/s12916-024-03253-x (PMC10835995; doi:10.1186/s12916-024-03253-x)
Supplement: Supplementary file 7 — Additional file 7: Supplementary Material 7. Funnel plots. [file 12916_2024_3253_MOESM7_ESM.docx]

**SUPPLEMENTARY MATERIAL 7** Funnel plots.

**Fig. 1** Depressive symptoms.


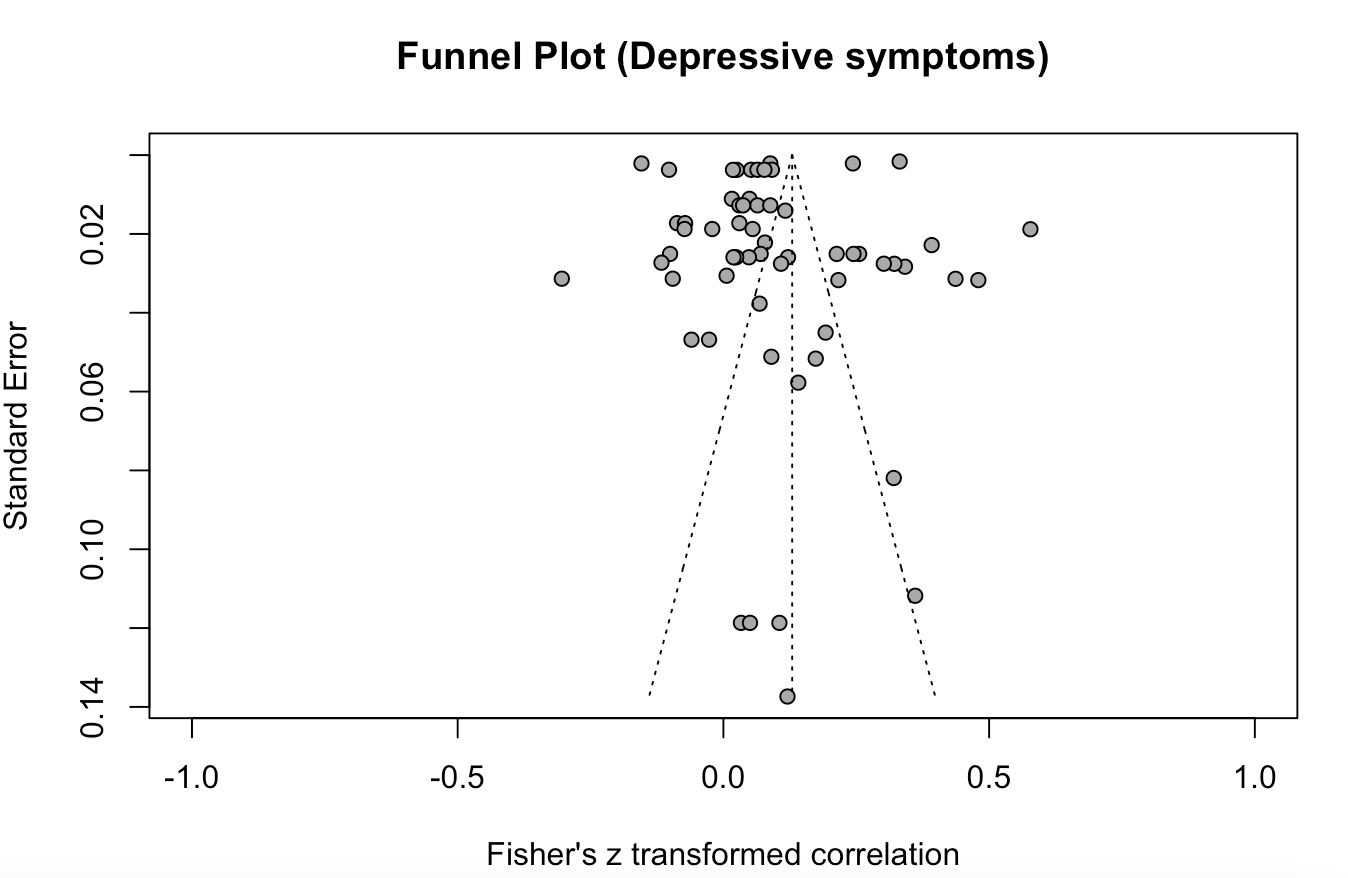


**Fig. 2** Anxiety symptoms.


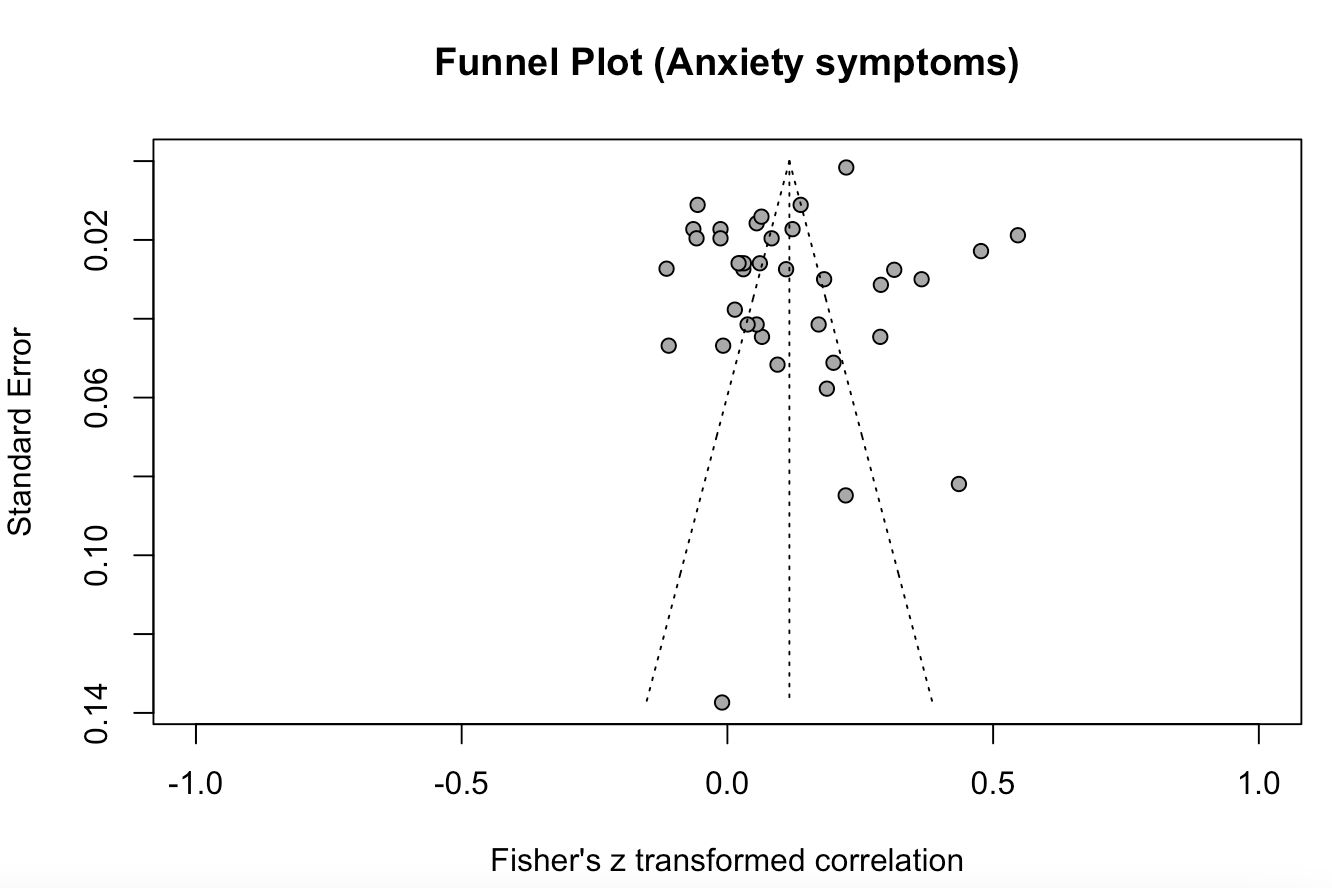


**Fig. 3** Posttraumatic stress disorder (PTSD) symptoms.


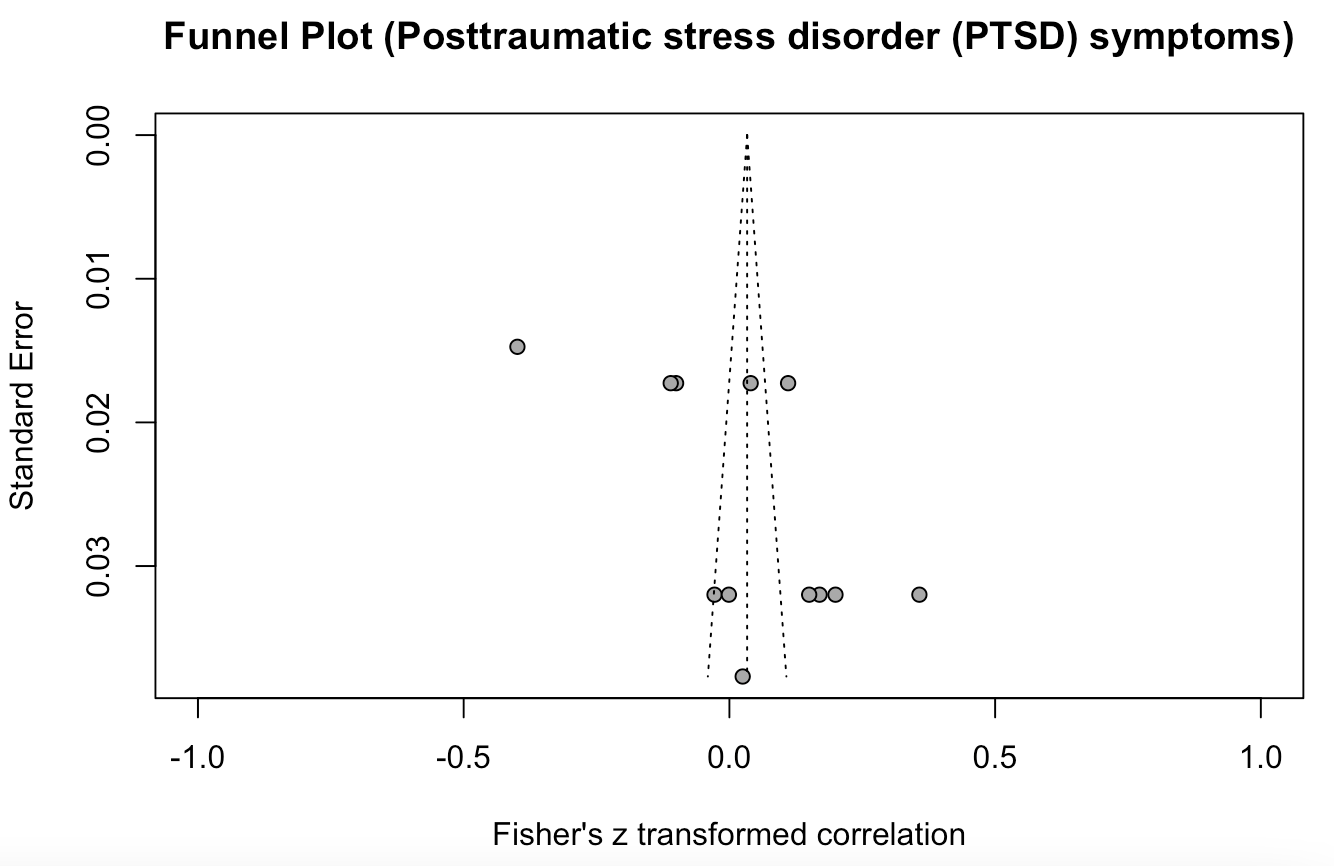


**Fig. 4** Depressive and anxiety symptoms.


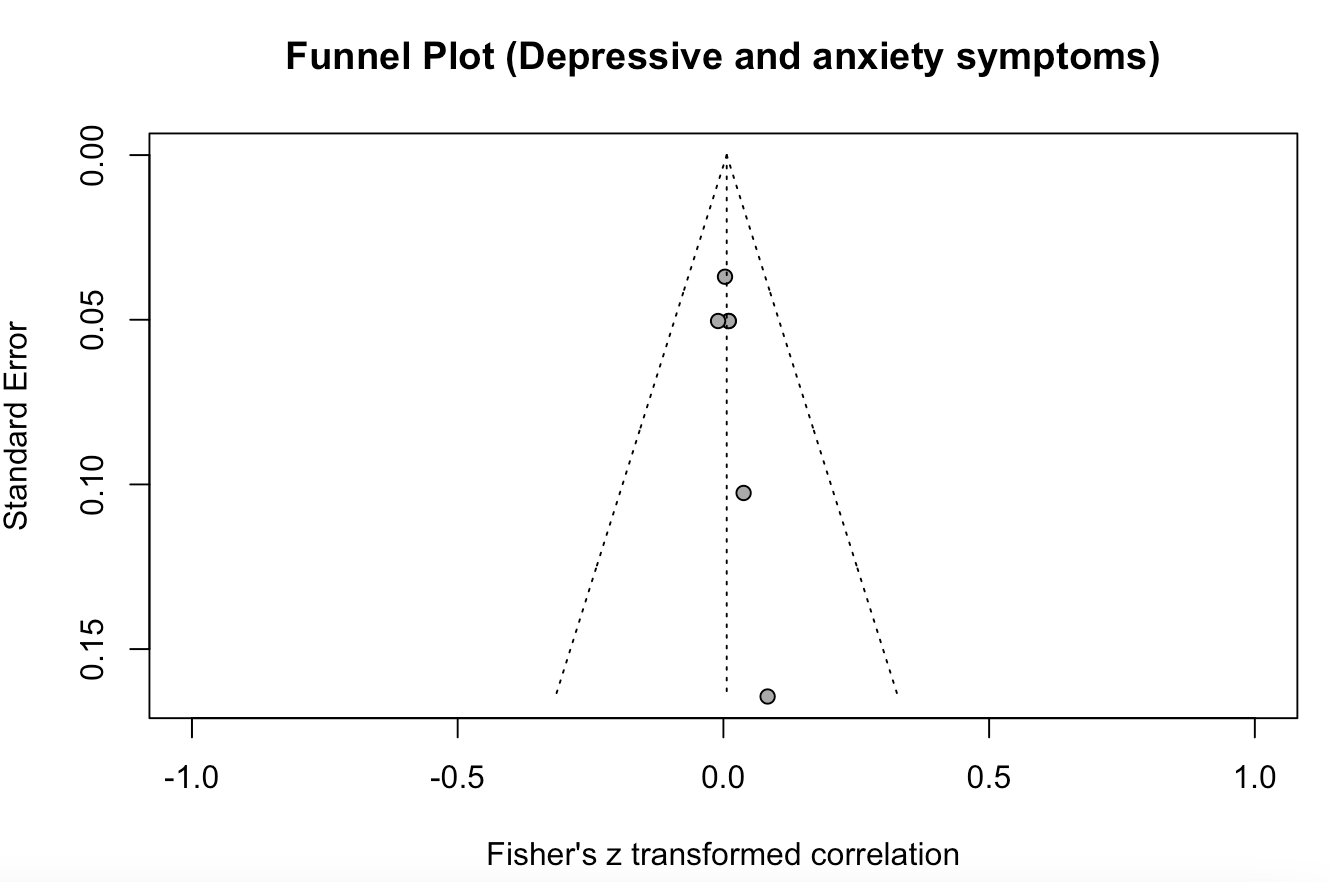


**Fig. 5** General psychological distress.

**
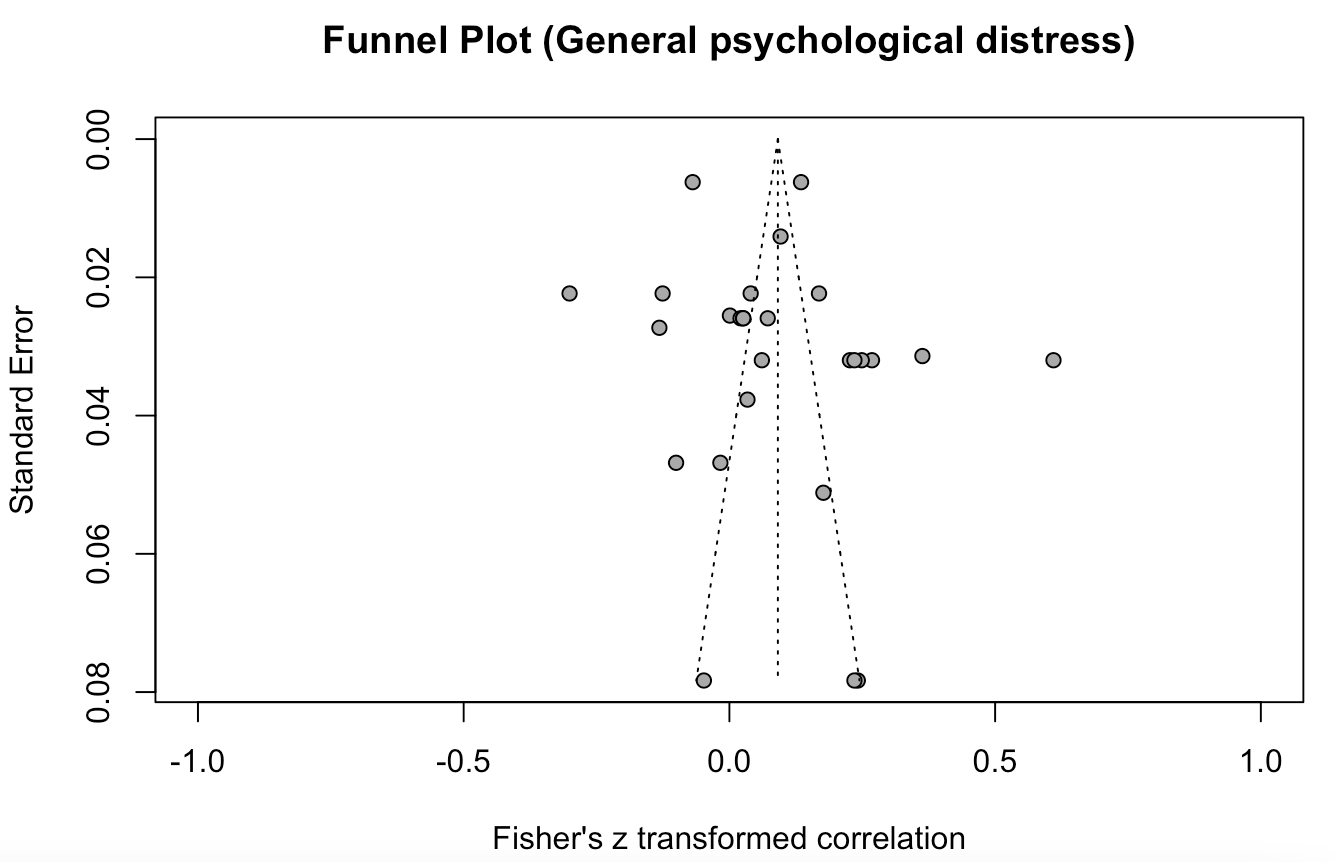
**
