# Supplementary material for: Daily routine disruptions and psychiatric symptoms amid COVID-19: a systematic review and meta-analysis of data from 0.9 million individuals in 32 countries
Source: BMC Med. 2024 Feb 2;22:49. doi: 10.1186/s12916-024-03253-x (PMC10835995; doi:10.1186/s12916-024-03253-x)
Supplement: Supplementary file 8 — Additional file 8: Supplementary Material 8. Publication bias statistics (53 studies, 51 independent samples). [file 12916_2024_3253_MOESM8_ESM.docx]

**SUPPLEMENTARY MATERIAL 8** Publication bias statistics (53 studies, 51 independent samples).

| **Outcome** | ***k*** | **Classic fail-safe *N*** | **Studies trimmed *N*** | **Egger’s regression intercept [95% CI]** | ***t*** | ***p*** |
| --- | --- | --- | --- | --- | --- | --- |
| Depressive symptoms | 62 | 3594990 | 25 | -2.84 [-22.41; 16.72] | -0.29 | 0.78 |
| **Anxiety symptoms** | **38** | **33000** | **12** | **-4.54 [-7.34; -1.74]** | **-3.18** | **<0.01** |
| **Post-traumatic stress disorder (PTSD) symptoms** | **12** | **0** | **4** | **16.93 [2.96; 30.90]** | **2.38** | **0.04** |
| **Depressive and anxiety symptoms** | **6** | **0** | **2** | **0.59 [0.20; 0.97]** | **3.00** | **0.04** |
| General psychological distress | 27 | 2719 | 5 | 2.28 [-2.25; 6.80] | 0.99 | 0.33 |

Notes. *k* = Number of effect sizes. Bold texts indicate significant results. Results of publication bias analyses for outcomes with few comparisons (*k* < 10) should be interpreted with caution.
